# Allostatic-Interoceptive Overload in Frontotemporal Dementia

**DOI:** 10.1016/j.biopsych.2022.02.955

**Published:** 2022-02-24

**Authors:** Agustina Birba, Hernando Santamaría-García, Pavel Prado, Josefina Cruzat, Agustín Sainz Ballesteros, Agustina Legaz, Sol Fittipaldi, Claudia Duran-Aniotz, Andrea Slachevsky, Rodrigo Santibañez, Mariano Sigman, Adolfo M. García, Robert Whelan, Sebastián Moguilner, Agustín Ibáñez

**Affiliations:** Latin American Brain Health Institute (AB, PP, JC, ASB, CD-A, SM, AI); Center for Social and Cognitive Neuroscience (CD-A), School of Psychology, Universidad Adolfo Ibáñez; Center for Geroscience, Brain Health and Metabolism (AS); Neuropsychology and Clinical Neuroscience Laboratory (AS), Physiopathology Department, Institute of Biomedical Sciences; Neurology Service (RS), Hospital Dr. Sótero del Río; Neurology Department (RS), Pontificia Universidad Católica de Chile; Departamento de Lingüística y Literatura (AMG), Facultad de Humanidades, Universidad de Santiago de Chile; Memory and Neuropsychiatric Clinic (AS), Neurology Department, Hospital del Salvador and Faculty of Medicine, University of Chile; Servicio de Neurología (AS), Departamento de Medicina, Clínica Alemana-Universidad del Desarrollo, Santiago, Chile; National Scientific and Technical Research Council (AB, AL, SF, MS, AMG, SM, AI); Cognitive Neuroscience Center (AB, AL, SF, AMG, SM, AI), Universidad de San Andrés; and Laboratorio de Neurociencia (MS), Universidad Torcuato Di Tella, Buenos Aires, Argentina; PhD Neuroscience Program (HS-G), Physiology and Psychiatry Departments, Pontificia Universidad Javeriana; and Memory and Cognition Center Intellectus (HS-G,), Hospital Universitario San Ignacio, Bogotá, Colombia; Global Brain Health Institute (H-SG, AMG, RW, SM, AI), University of California San Francisco, San Francisco, California, and Trinity College Dublin, Dublin, Ireland; Trinity College Institute of Neuroscience (RW, AI), Trinity College Dublin, Dublin, Ireland; Facultad de Lenguas y Educación (MS), Universidad Nebrija, Madrid, Spain; and Department of Neurology (SM), Massachusetts General Hospital and Harvard Medical School, Boston, Massachusetts

## Abstract

**BACKGROUND:**

The predictive coding theory of allostatic-interoceptive load states that brain networks mediating autonomic regulation and interoceptive-exteroceptive balance regulate the internal milieu to anticipate future needs and environmental demands. These functions seem to be distinctly compromised in behavioral variant frontotemporal dementia (bvFTD), including alterations of the allostatic-interoceptive network (AIN). Here, we hypothesize that bvFTD is typified by an allostatic-interoceptive overload.

**METHODS:**

We assessed resting-state heartbeat evoked potential (rsHEP) modulation as well as its behavioral and multimodal neuroimaging correlates in patients with bvFTD relative to healthy control subjects and patients with Alzheimer’s disease (*N* = 94). We measured 1) resting-state electroencephalography (to assess the rsHEP, prompted by visceral inputs and modulated by internal body sensing), 2) associations between rsHEP and its neural generators (source location), 3) cognitive disturbances (cognitive state, executive functions, facial emotion recognition), 4) brain atrophy, and 5) resting-state functional magnetic resonance imaging functional connectivity (AIN vs. control networks).

**RESULTS:**

Relative to healthy control subjects and patients with Alzheimer’s disease, patients with bvFTD presented more negative rsHEP amplitudes with sources in critical hubs of the AIN (insula, amygdala, somatosensory cortex, hippocampus, anterior cingulate cortex). This exacerbated rsHEP modulation selectively predicted the patients’ cognitive profile (including cognitive decline, executive dysfunction, and emotional impairments). In addition, increased rsHEP modulation in bvFTD was associated with decreased brain volume and connectivity of the AIN. Machine learning results confirmed AIN specificity in predicting the bvFTD group.

**CONCLUSIONS:**

Altogether, these results suggest that bvFTD may be characterized by an allostatic-interoceptive overload manifested in ongoing electrophysiological markers, brain atrophy, functional networks, and cognition.

Predictive coding theories propose that the brain continuously updates an internal model of the world to anticipate the organism’s future states and its responses to external demands (1–3). This process of continuous adjustment to anticipate environmental demands is known as allostasis; the cost of maintaining allostasis is known as allostatic load (4). Within this framework, the allostatic-interoceptive load (1,2,5–7) refers to internal body states (e.g., moment-to-moment visceral signals) that modulate the response to environmental demands. Allostatic-interoceptive load has been empirically linked to changes in bodily autonomic regulation (2,8,9). An allostatic-interoceptive network (AIN) has been identified in the human brain (2). The AIN involves the intersection of two large-scale intrinsic networks, the salience network (SN) (bilateral ventral and dorsal anterior insula, anterior cingulate cortex, ventral striatum, thalamus, amygdala, hypothalamus, and brainstem) and the default mode network (DMN) (bilateral angular gyrus, precuneus, hippocampus, and medial prefrontal cortices) (2). The AIN-specific connections of each network involve the dorsal mid to posterior insula (primary interoceptive cortex), limbic nodes, motor and premotor cortices (visceromotor control), and other frontotemporoparietal connections (2). Thus, the predictive coding theory of allostatic-interoceptive load (2,8) proposed that visceral signals regulate responses to external stimuli within the AIN (2).

Allostatic-interoceptive overload involves an imbalance between aberrant interoceptive signals and the responses to environmental demands (4). This imbalance of brain-body communication has been associated with pathophysiology [including autonomic outflow, abnormal stress responses, cognitive dysfunction, and behavioral disturbances (4,10)]. The heartbeat evoked potential (HEP), a brain response triggered by visceral signals and modulated internal body sensing (11), can be considered a marker of allostatic-interoceptive load. Across cognitive domains, the moment-to-moment body internal states tracked with HEP modulate the brain and cognitive responses to external stimuli (12–17). Source analyses of HEP (12,18) and its neuroimaging correlates (19,20) are concordant with the allostatic-interoceptive brain regions as described above. Although HEP has been traditionally used in heartbeat detection tasks, recent reports have shown HEP modulation in resting-state conditions (rsHEP) and during noncardiac monitoring studies (12,21). In particular, increased rsHEP has been associated with the hypervigilance to interoceptive signals linked to allostatic overload (21,22).

Thus, according to the predictive coding framework, allostatic-interoceptive overload can be 1) indexed by increased rsHEP modulations, 2) associated with specific, relevant cognitive dysfunctions, and 3) linked to brain anatomy and connectivity of the AIN. Here, we tested these three predictions in behavioral variant frontotemporal dementia (bvFTD). This is the most prevalent form of frontotemporal lobar degeneration, characterized by early personality changes, behavioral inappropriateness, and frontotemporoinsular neurodegeneration (23,24). Several sources of evidence suggest that bvFTD may be associated with an allostatic-interoceptive overload, which we describe below.

First, body autonomic dysregulation is pervasive in bvFTD (13,25), including impaired skin conductance (26), cardiac vagal tone (27), heart rate, resting, and total energy expenditure (28). Atrophy and dysfunction in the left frontoinsula in bvFTD are deleterious for parasympathetic activity (27). Although autonomic imbalance and dysregulated behaviors involve increased rsHEP modulation in other conditions (22,29–33), this has not been tested in bvFTD. However, interoceptive impairments and their brain correlates have been identified in bvFTD (34,35). Moreover, these interoceptive impairments in bvFTD have been associated with deficits in recognizing others’ emotions (13). Thus, previous research suggests that rsHEP should be impaired in bvFTD and that this impairment will be linked to specific deficits in cognitive-emotional responses to stimuli.

Second, the sociocognitive dysfunctions of bvFTD may be linked to interoceptive dysregulation. Patients with bvFTD typically exhibit impairments in the cognitive state (CS), defined as individual performance across core general functions, such as attentional, memory, and visuospatial domains; executive functions (EFs); and facial emotion recognition (FER) (36). Although not yet linked to allostatic-interoceptive overload in bvFTD, these sociocognitive processes are plausibly affected by such overload (37). Moreover, theoretical accounts (24) and metanalytic evidence (38) suggest that cognitive processes usually impaired in bvFTD (social cognition, emotion recognition, and interoception) are interlinked. Patients with bvFTD have poorer responses to external demands (disinhibition, disruptive or inappropriate behaviors) [for a review, see (39)] and exacerbated reactions in social settings [i.e., increased experience of envy and schadenfreude (40); violation of social norms (41)]. These sociocognitive inappropriate responses in bvFTD have been theoretically—but not empirically—linked to allostatic-interoceptive overload (24,42–45). Beyond these antecedents, the specific association of increased rsHEP and sociocognitive dysfunctions in bvFTD has not been directly tested.

Third, most of the allostatic-interoceptive brain regions are affected by neurodegeneration in bvFTD (46,47). Autonomic disruption in bvFTD depends on the integrity of insular networks (48). The core hubs of the AIN (2) comprising specific subhubs of the SN and DMN (2) are impaired in bvFTD (49–54). The SN has been associated with interoceptive deficits (34,35) and impairments on interoceptive priming of recognition of other’s emotion (13) in bvFTD. However, although the brain atrophy and connectivity impairment in bvFTD seems to overlap with the AIN, no previous work has examined the potential aberrant rsHEP activity and its specific association with the AIN.

The extant work described above indicates a hypothetical allostatic-interoceptive overload in bvFTD, which remains to be tested empirically. To assess this, we measured rsHEP modulation and its sociocognitive and multimodal neuroimaging correlates in patients with bvFTD relative to healthy control (HC) subjects and patients with Alzheimer’s disease (AD). Patients with AD were included as a control neurodegenerative disease to test the selectivity of the hypothesized impairments in bvFTD. We hypothesized that rsHEP in bvFTD would be 1) selectively increased, 2) associated with sociocognitive dysfunctions, and 3) linked explicitly to atrophy and connectivity of the AIN. Our hypothesis leads to three sets of specific predictions. First, we anticipated a selective exacerbated rsHEP (increased amplitude modulation with sources in allostatic-interoceptive regions) in bvFTD compared with HC and AD groups. Second, even though both AD and bvFTD commonly present with cognitive, EF, and emotion recognition deficits, we predicted specific associations of rsHEP overload and impairments in these measures only in bvFTD. Finally, we anticipated that rsHEP modulation in bvFTD would be selectively related deficits in multimodal neuroimaging signatures (atrophy of allostatic-interoceptive regions and reduced connectivity of AIN in comparison with other control brain networks).

## METHODS AND MATERIALS

### Participants

The study comprised 94 participants: 19 patients with bvFTD, 33 patients with AD, and 42 HC subjects. This sample size reached an adequate statistical power (0.96) to address our predictions (see section 1 of the Supplement). Patients were diagnosed by expert neurologists following current criteria for probable bvFTD (55), and National Institute of Neurological and Communicative Disorders and Stroke–Alzheimer’s Disease and Related Disorders Association clinical criteria (56,57) for AD. Recruitment and diagnosis were conducted in clinical centers by a multidisciplinary team as part of an ongoing multicentric protocol (15,58). Diagnoses were supported by extensive examinations (14,23,59,60), in line with the Multi-Partner Consortium to Expand Dementia Research in Latin America standardized protocol (61,62). The CS, EF, and FER tasks were not considered as diagnostic criteria. Patients with bvFTD were in the mild stage of the disease and presented ventromedial compromise, associated with executive impairments (63–66). No patient reported a history of other neurologic disorders, psychiatric conditions, primary language deficits, or substance abuse. All participants provided written informed consent pursuant to the Declaration of Helsinki. The study was approved by the Ethics Committees of the involved institutions. Demographic features of participants and their neuropsychological assessment are provided in Table 1.

### Experimental Protocol

**Figure 1.**
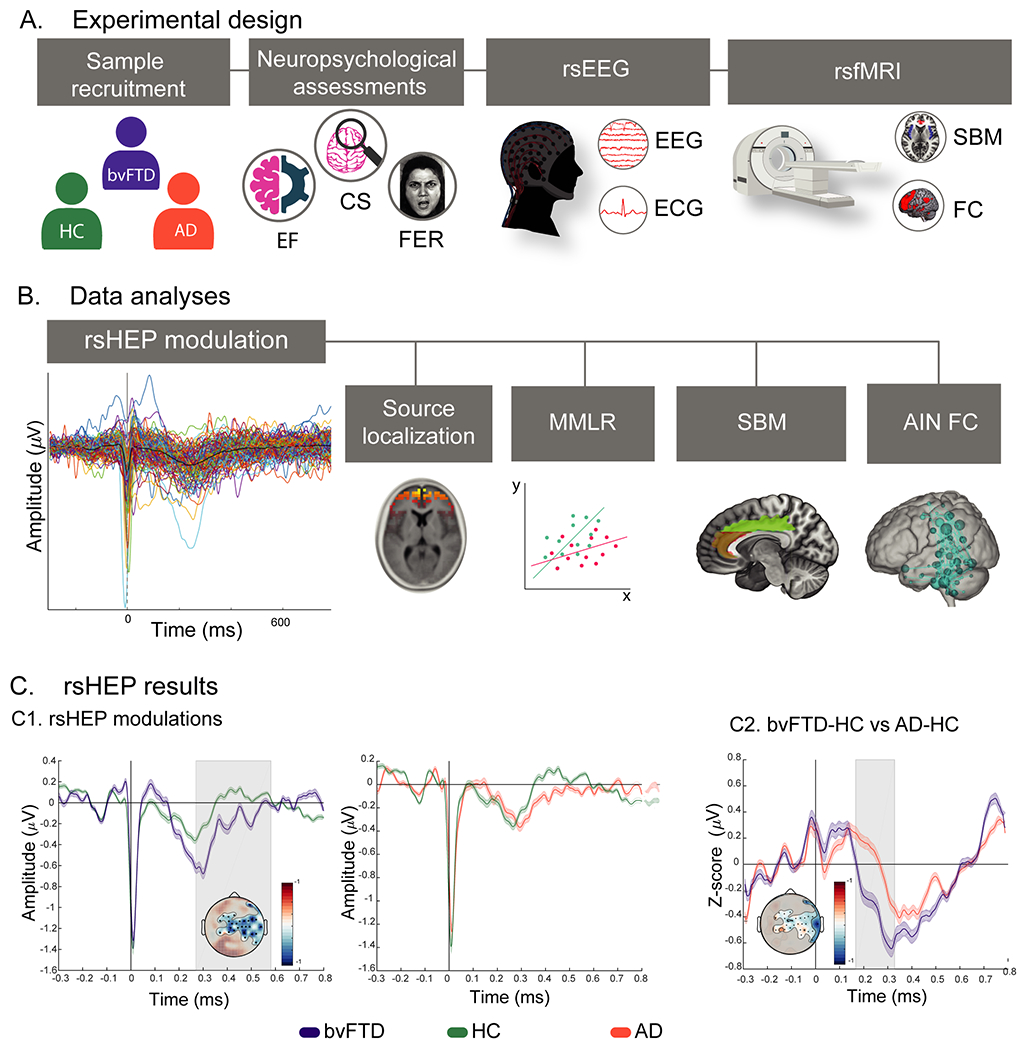
Experimental design and high-density electroencephalography (hdEEG) results. **(A)** Experimental design. Participants completed a neuropsychological assessment evaluating cognitive state (CS), executive functions (EFs), and facial emotion recognition (FER). The protocol involved a 10-minute resting-state session while high-density EEG signals were recorded and a resting-state magnetic resonance imaging (MRI) and functional MRI (fMRI) session. **(B)** Data analyses. First the resting-state heartbeat evoked potential (rsHEP) from the high-density EEG signal and its sociocognitive (CS, EF, and FER) and multimodal neuroimaging correlates (source localization, surface-based morphometry [SBM] and functional connectivity [FC]-fMRI analyses) were calculated. **(C)** rsHEP results. **(C1)** rsHEP modulations during resting-state. Left: Healthy control (HC) subjects (green line) vs. behavioral variant frontotemporal dementia (bvFTD) (violet line). Right: HC (green line) vs. Alzheimer’s disease (AD) (pink line). Gray shaded boxes show statistically significant differences at *p* = .05 cluster corrected (from 290 to 600 ms). Scalp topographies show the significant electrodes of the cluster and the differences in amplitude (microvolts) between rsHEP at 400 ms. **(C2)** Subtraction of the mean rsHEP modulations between bvFTD and HC (violet line) and between AD and HC (pink line) in the cluster significant electrodes. Gray shaded boxes show statistically significant differences at *p* = .05 false discovery rate–corrected (between 190 and 310 ms). Scalp topographies show the differences in amplitude (microvolts) at 250 ms and the electrodes used for the analysis. AIN, allostatic-interoceptive network; ECG, electrocardiogram; MMLR, multivariate multiple regression; rsEEG, resting-state EEG; rsfMRI, resting-state fMRI.

Figure 1A describes the experimental protocol.

### Neuropsychological Assessment

CS, EF, and FER assessment are detailed in section 8 of the Supplement.

### High-Density Electroencephalography Methods

#### Acquisition and Signal Preprocessing.

We obtained high-density electroencephalography (EEG) signals during a 10-minute resting-state protocol detailed in section 3 of the Supplement (67). To examine HEP, we segmented the continuous EEG signal into epochs from −300 to 800 ms around the R-wave peak and baseline-corrected relative to −300 ms time window preceding the heartbeat (15,17,40,68–70). Noisy epochs were rejected using the criteria of trials that exceeded a threshold of 2.5 standard deviations from the mean probability distribution calculated from all trials and then measuring probability distribution kurtosis (71). The percentage of final trials was similar across groups (see section 3 of the Supplement). Low drifts were removed by linear trend corrections (72). Trials were averaged across subjects for group comparisons.

#### Spatiotemporal Clustering.

The significance of differences in HEP modulation between groups was tested using a cluster-corrected permutation test (73), implemented in the FieldTrip toolbox (74). The *t* values that exceeded a threshold of *p* < .05 (two-tailed) were clustered based on spatiotemporal adjacency. Each resulting cluster was assigned cluster-level statistics corresponding to the sum of the *t* values of the samples belonging to that cluster. A minimum of 10 neighboring electrodes was required to pass this threshold and form a robust cluster (75). We assessed the significance of a spatiotemporal cluster identified above. This procedure was repeated 5000 times, with recombination and randomized resampling of the subjectwise averages before each repetition using the Monte Carlo method (76). See full details in the Supplement.

#### Source Localization.

Whole-brain activation maps were estimated for each subject for each of the 564 scalp voltage distributions comprising the time window defined for rsHEP analysis (−300 to 800 ms around the R-wave of the heartbeat). As in spatiotemporal clustering analyses of HEP for scalp data, we calculated normalized *z* scores for comparison of bvFTD and AD using the parameters (mean and SD) derived from the respective control group. Activation maps were obtained using the Bayesian model approach of the EEG inverse problem (77) implemented in a Source Localizer (78). Bayesian model approach addresses the problem of model uncertainty that arises when particular data (EEG scalp distribution in this case) is explained by different models such as primary current densities inside the brain. In this case, a set of models is defined based on anatomical constraints, each model consisting of a particular combination of anatomical compartments to which the generation of primary current densities is restricted (79). Bayesian model approach allows for the accurate estimation of deep EEG sources and results in primary current density maps with lower localization error and higher resolution than those obtained with traditional source analysis methods (77,80). See full details in section 3 of the Supplement.

#### Prediction of Behavior With rsHEP.

To evaluate the relationship between neurovisceral markers of allostatic load (rsHEP) and cognitive deficits in bvFTD, we ran a multivariate multiple linear regression [MMLR (81)] considering rsHEP modulations as predictor and the three neuropsychological variables (CS, EF, FER) as outcomes. See section 8 of the Supplement for the full procedure.

### Neuroimaging Methods

#### Data Acquisition.

Magnetic resonance imaging (MRI)/functional MRI acquisition and preprocessing steps are reported following Organization for Human Brain Mapping recommendations (82,83) (Figure 1A). We acquired three-dimensional volumetric and 10-minute-long resting-state MRI sequences. As in the case of high-density EEG recordings, participants were instructed not to think about anything in particular while remaining still, awake, and with eyes closed (17,84). For details, see section 4 of the Supplement.

#### Surface-Based Morphometry Preprocessing and Analysis.

We assessed whether rsHEP modulation correlates with the volume and cortical thickness of key allostatic-interoceptive regions. We processed all T1 images via surface-based morphometry on the FreeSurfer software suite (version 6.0 https://surfer.nmr.mgh.harvard.edu/). See section 4 of the Supplement for details.

#### Preprocessing of Functional MRI Data for Resting-State Functional Connectivity Analysis.

Images were then preprocessed using the Data Processing Assistant for Resting-State fMRI (DPARSF version 2.3) (85) as detailed in section 4 of the Supplement.

#### Analysis of Resting-State Functional Connectivity Functional MRI Data.

We evaluated associations between rsHEP modulations and functional connectivity (FC) patterns. We implemented a seed analysis to examine the associations between HEP modulation and FC of the AIN (1) (Figure 1B). To test the specificity of our predictions for these networks, we also examined the associations of rsHEP modulation with the connectivity of five additional functional networks: the SN, executive network (EN), DMN, visual network (VN), and motor network (MN). By testing the specificity of rsHEP as a marker of interoceptive allostatic overload in bvFTD, we expected nonsignificant associations between rsHEP modulations and the control networks. See section 4 of the Supplement for details on analysis.

## RESULTS

### High-Density EEG Results

#### rsHEP Modulations.

rsHEP comparisons between patients with bvFTD and HC subjects revealed a significant cluster over right centrotemporal regions in a window of 290 to 600 ms after R-peak period (*t*-sum = −6141.61, cluster-level *p* = .004, *d* = 0.78 corrected for multiple comparisons in space and time) (Figure 1C1, left panel), with its maximum *t* value at 400 ms. Specifically, patients with bvFTD presented a larger negative amplitude than HC subjects, indexing greater rsHEP modulation. The results were not biased by the difference in sample size across groups (see section 6 of the Supplement). No significant clusters were observed between patients with AD and HC subjects (*t*-sum = −2481.15, cluster-level *p* = .08) (Figure 1C1, right panel).

To control for potential confounding differences between patients with bvFTD and patients with AD, we compared their respective normalized (*z*-scored) mean rsHEP modulations in the cluster differentiating patients with bvFTD from HC subjects. The difference was higher for patients with bvFTD than for patients with AD between 190 and 310 ms (*p* < .05, false discovery rate [FDR]–corrected, *d* = 0.50) (Figure 1C2). Overall, the results show that rsHEP is increased in patients with bvFTD relative to both control groups (HC and AD).

#### Source Space of rsHEP.

The source localization analyses of rsHEP in patients with bvFTD revealed statistically significant activation in most of the AIN hubs (crucially, the insula, anterior cingulate cortex, and amygdala) (permutation test: mean vs. 0 contrast, *p* < .05 FDR-corrected); see section 7.1 of the Supplement. In the same line, HC subjects and patients with AD also presented a distributed brain activation within the AIN (permutation test: mean vs. 0 contrast, *p* < .05 FDR-corrected) (see sections 7.2 and 7.3 of the Supplement, respectively).

**Figure 2.**
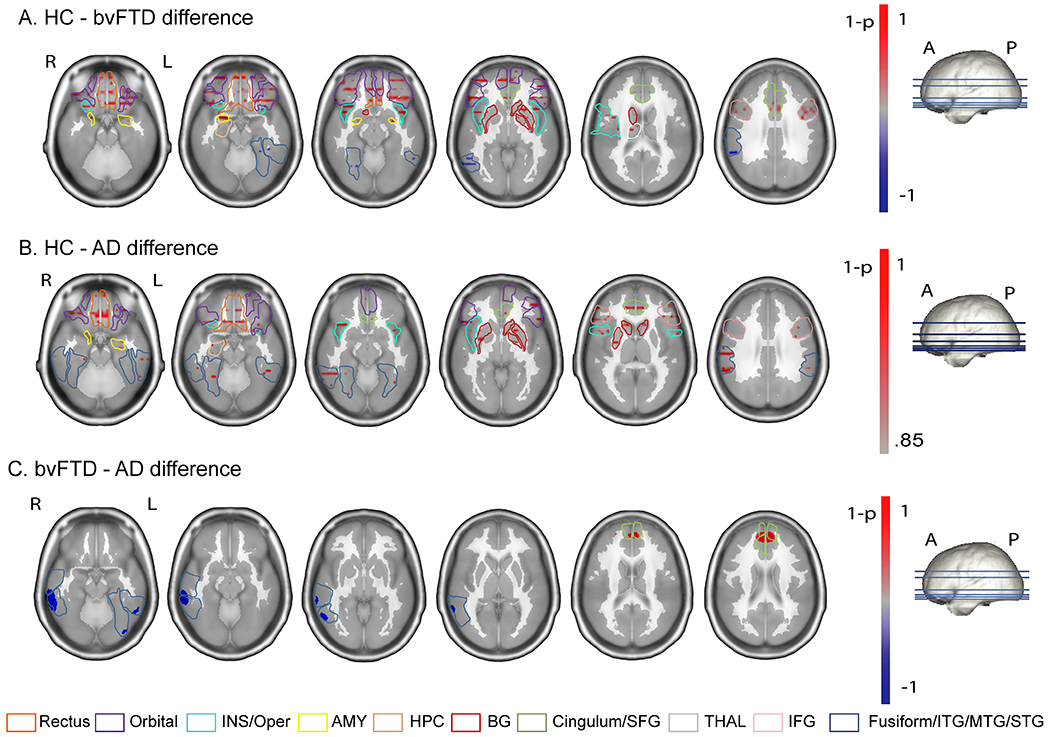
Source localization results. Activation maps were obtained using the Bayesian model approach (77) of the electroencephalography inverse problem [implemented in Neuronic Source Localizer (77,78)]. **(A)** Subtraction of the mean activation maps between healthy control (HC) subjects and patients with behavioral variant frontotemporal dementia (bvFTD). **(B)** Subtraction of the mean activation maps between HC subjects and patients with Alzheimer’s disease (AD). Bayesian model approach images were visualized using the software Neuronic Tomography Viewer and segmented with the AAL atlas (140). **(C)** Subtraction of the mean activation maps between AD (*z*-scored) and bvFTD (*z*-scored). A, anterior; AMY, amygdala; BG, basal ganglia; HPC, hippocampus; INS, insula; ITG, inferior temporal gyrus; L, left; P, posterior; R, right; STS, superior temporal sulcus; THAL, thalamus.

Compared with HC subjects, patients with bvFTD showed significantly lower activation in relevant interoceptive hubs, including the insula, anterior cingulate cortex, opercular region, and thalamus (permutation test, HC > bvFTD contrast: *p* < .05, FDR-corrected). The compromise of these primary nodes was accompanied by hyperactivation of temporal regions of the AIN (permutation test, HC < bvFTD contrast: *p* < .05, FDR-corrected) (Figure 2A) (see section 7.4 of the Supplement. The aforementioned regions included the inferior temporal gyrus/superior temporal sulcus and supramarginal and fusiform cortices, which are relevant for visuomotor control, sensory integration, and stress regulation. In contrast, patients with AD showed lower activation in frontal and temporal cortices (permutation test, HC > AD contrast: *p* < .05, FDR-corrected) (Figure 2B) (see section 7.5 of the Supplement and presented no exacerbated activity (permutation test, HC < AD contrast: all *p* values > .05, FDR-corrected). Regarding AD and bvFTD comparisons, patients with bvFTD had lower activation in the superior frontal medial gyrus and anterior cingulum (permutation test, AD > bvFTD contrast: *p* < .05, FDR-corrected) (Figure 2C) (see section 7.6 of the Supplement) and hyperactivation in the inferior and medial temporal areas and the inferior lingual and occipital cortices (permutation test, bvFTD > AD contrast: all *p* values > .05, FDR-corrected) (Figure 2C) (see section 7.6 of the Supplement. Thus, the rsHEP source space results indicate a combination of reduced (insula, anterior cingulate cortex, opercular region, and thalamus) and overactive (inferior temporal gyrus/superior temporal sulcus, supramarginal and fusiform cortices) regions of the AIN only in patients with bvFTD. These results were not biased by the difference in sample size across groups (see section 7.7 of the Supplement).

#### bvFTD Specific Associations of rsHEP and Multiple Sociocognitive Measures.

We evaluated the extent to which rsHEP in bvFTD was related to cognitive deficits by running an MMLR (59) considering rsHEP modulations as the predictor variable and the three neuropsychological measures (CS, EF, FER) as outcomes. We compared the rsHEP and cognitive associations in the three groups (bvFTD, AD, and HC). The MMLR model included CS, EF, and FER scores (dependent variables) and the interaction between the rsHEP modulation (area under the curve) and group (dummy variable: bvFTD-HC-AD) as predictors. We expected that rsHEP would explain the sociocognitive deficits only in bvFTD. rsHEP modulation significantly predicted the three neuropsychological scores in patients with bvFTD, as evidenced by the significant interaction between rsHEP and the bvFTD group (CS: β = 0.04, *p* = .05; EF: β = 0.06, *p* = .01, FER: β = 0.06, *p* = .01) (for full results, see Table 2 and Figure 2A) but not in patients with AD or HC subjects. The larger the negative rsHEP modulation, the higher the deficit of patients with bvFTD in multimodal behavioral performance, including CS, EF, and FER. Additional MMLR analysis with bvFTD and AD groups separately showed the same results (see section 8 of the Supplement) and were not biased by the difference in sample size across groups (see section 8.1 of the Supplement). Furthermore, given the importance of these results, we have conducted multiple checks to assess their robustness (see section 8.2 of the Supplement). In particular, we replicated the previous result with alternative bootstrapping methods and confirmed that low sample sizes and the potential nonlinear data do not explain these significant results.

### Neuroimaging Results

#### Associations Between Brain Structure and rsHEP Modulation.

**Figure 3.**
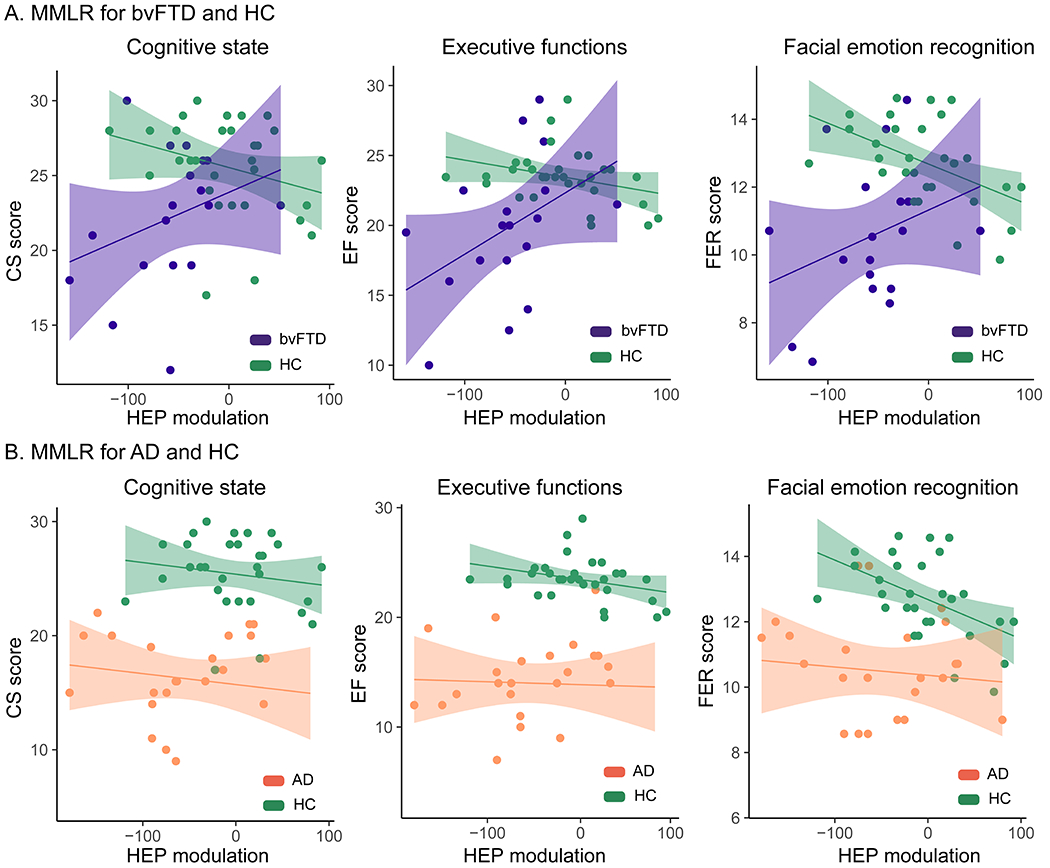
Multivariate multiple linear regression (MMLR) models. **(A)** Association between resting-state heartbeat evoked potential (rsHEP) modulation and neuropsychological performance for behavioral variant frontotemporal dementia (bvFTD) and healthy control (HC) subjects. Significant interactions (*p* < .05) between rsHEP modulation and group were found for the cognitive state (CS) (right), executive functions (EFs) (middle), and facial emotion recognition (FER) (left) scores, evidencing that only in bvFTD, the larger the negative rsHEP modulation (more negative values), the more increased the multimodal behavioral impairment. **(B)** Association between rsHEP modulation and neuropsychological performance for Alzheimer’s disease (AD) and HC subjects. Patients with AD were outperformed in the three neuropsychological tests by HC subjects, independently of the rsHEP modulation. For estimates and statistical details, see Table 2.

**Figure 4.**
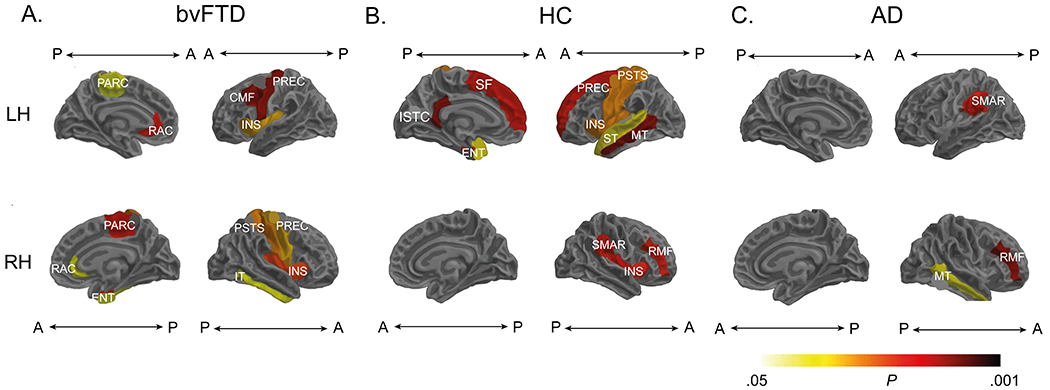
Associations between resting-state heartbeat evoked potential (rsHEP) modulation and whole-brain structure. **(A)** The behavioral variant frontotemporal dementia (bvFTD) group showed significant correlations of rsHEP modulation on core allostatic-interoceptive regions (anterior cingulate, bilateral insula, inferior temporal). **(B)** Healthy control (HC) group analyses revealed significant associations between rsHEP modulation and the cortical structure of allostatic-interoceptive regions (*p* < .05, false discovery rate–corrected). **(C)** The Alzheimer’s disease (AD) group exhibited positive association of rsHEP modulations with the cortical structure of the left middle temporal gyrus, right rostral middle frontal gyrus, and left supramarginal gyrus. Cortical structure was obtained via surface-based morphometry. Results are presented using Desikan-Killiany cortical atlas (141). For structural association details, see Supplement (bvFTD Specific Associations of rsHEP and Multiple Sociocognitive Measures). A, anterior; CMF, caudal middle frontal; ENT, entorhinal; INS, insula; ISTC, isthmus cingulate; IT, inferior temporal gyrus; LH, left hemisphere; MT, middle temporal gyrus; P, posterior; PARC, paracentral lobule; PREC, precentral; PSTS, postcentral; RAC, rostral anterior cingulate; RH, right hemisphere; RMF, rostral middle frontal; SF, superior frontal; SMAR, supramarginal gyrus; ST, superior temporal gyrus.

We assessed whether rsHEP modulation was related to the volume and cortical thickness of AIN regions. For patients with bvFTD, this modulation was positively correlated with the volume and cortical thickness of the bilateral insula, right amygdala, and bilateral anterior cingulate (*p* < .05, whole-brain FDR-corrected) (Figure 3B) (see section 8.1 of the Supplement). In the same vein, the HC group showed significant positive associations (*p* < .05, whole-brain FDR-corrected) between rsHEP modulation and the volume and cortical thickness of the bilateral insula and the left amygdala (Figure 4A) (see section 9.2 of the Supplement), among other regions (right superior and middle temporal gyrus). Finally, rsHEP modulation of the AD group correlated with the structure of the disease-atrophied regions, such as the middle temporal gyrus, left supramarginal gyrus, and middle frontal gyrus (Figure 3C) (see section 9.3 of the Supplement). Thus, rsHEP modulations, particularly in patients with bvFTD (and HC subjects) were related with anatomical integrity of key AIN regions. These results were not biased by the difference in sample size across groups (see section 9.4 of the Supplement).

#### Association of rsHEP With FC of AIN and Control Networks.

**Figure 5.**
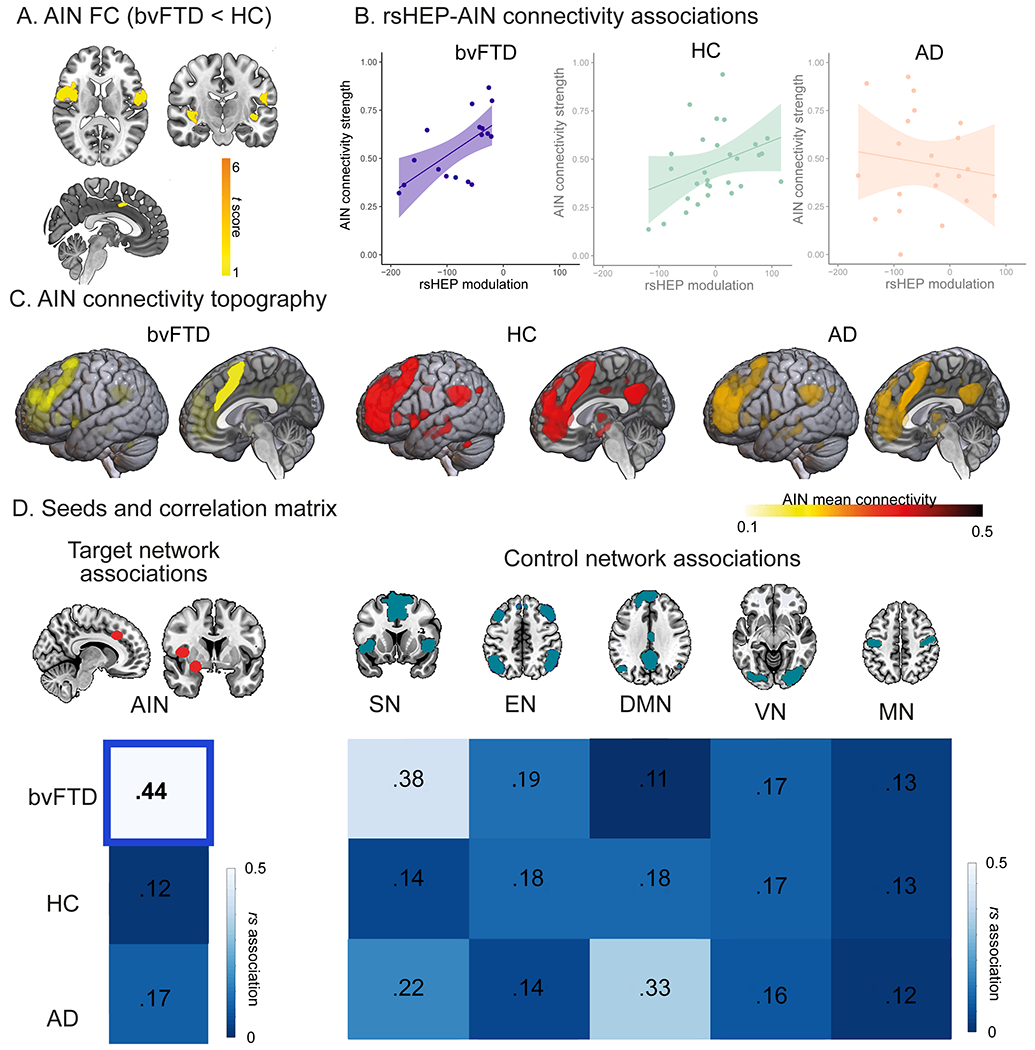
The resting-state heartbeat evoked potential (rsHEP) modulation and functional connectivity (FC) of the allostatic-interoceptive network (AIN) and control networks. Seed analyses over different networks (false discovery rate–corrected) were performed to test the AIN impairments in behavioral variant frontotemporal dementia (bvFTD) and associations between rsHEP modulation and the FC of each network. **(A)** FC differences between bvFTD and healthy control (HC) subjects. Patients with bvFTD exhibited lower mean connectivity across the bilateral insula and amygdala and the right anterior cingulate cortex. (*p* < .05 false discovery rate–corrected). **(B)** rsHEP-AIN associations. Only bvFTD showed significant positive correlation (*r* = 0.44, *p*-false discovery rate = .01). **(B)** AIN connectivity topography. Lateral and sagittal views of intrinsic connectivity discovery maps depicting all voxels whose time course is correlated with the AIN seeds (right dorsal middle insula, right anterior mid-cingulate, and right dorsal amygdala). **(C)** Seeds and correlation matrices. Left: seed analysis and correlation matrix of the AIN matrix for all groups. Right: control networks (salience network [SN], executive network [EN], motor network [MN], visual network [VN], default mode network [DMN]) and correlation matrix across groups. Bold font indicates statistical significance. AD, Alzheimer’s disease.

We first ran seed analyses to compare the AIN between patients and HC subjects and then examined associations between rsHEP and the AIN as well as five control networks (SN, EN, DMN, VN, and MN). Compared with HC subjects, patients with bvFTD exhibited lower mean connectivity across specific AIN hubs (bilateral insula and amygdala and right anterior cingulate cortex) (*p* < .05, FDR-corrected). This pattern seemed distinctive of bvFTD, because the AD group did not show significant AIN hypoconnectivity compared with HC subjects (*p* > .05, FDR-corrected). Furthermore, for the bvFTD group, seed analysis revealed a significant positive association between connectivity of the AIN and rsHEP modulation: the larger the rsHEP modulation, the lower the connectivity of the AIN (*r* = 0.44, *p*-FDR = .01) (Figure 5A). Conversely, rsHEP modulation of both patients with AD and HC subjects exhibited no significant associations with connectivity of the AIN (all *p* values > .05, FDR-corrected) (Figure 5A). Finally, the specificity of the rsHEP-AIN association in bvFTD was confirmed by nonsignificant associations between rsHEP modulation and control networks across groups (including the SN, EN, DMN, VN, and MN; all *p* values > .05, FDR-corrected) (Figure 5C). These results were not biased by the difference in sample size across groups (see section 10.1 of the Supplement). Furthermore, we computed Bayes factors quantifying evidence for or against the presence of correlation between AIN connectivity and rsHEP (see section 10.2 of the Supplement)

#### Machine Learning Approach to Multimodal Allostatic-Interoceptive Features in bvFTD.

Our hypothesis suggests a multimodal (HEP sources, atrophy, and FC) allostatic-interoceptive overload impairment in bvFTD. To assess if these allostatic-interoceptive contributions were specific for the bvFTD group, we ran a machine learning algorithm with rsHEP sources, FC, and volumetric data as features to classify across the three groups. In short, for bvFTD discrimination—versus HC subjects and AD—the classificatory relevance was highest for the allostatic-interoceptive features (see section 11.0 of the Supplement).

## DISCUSSION

This work examined the hypothesis of bvFTD selectively presenting an increased rsHEP modulation linked to specific sociocognitive disfunctions, atrophy, and connectivity of the AIN. Altogether, our findings suggest that disparate physio-pathological processes and behavioral impairments constituting hallmarks of bvFTD could be better understood under a predictive coding framework of allostatic-interoceptive overload.

rsHEP modulation was exacerbated in patients with bvFTD but not in patients with AD or HC subjects. Such an effect was observed over right frontocentrotemporal topographies within the expected time window (200–800 ms). Increased rsHEP amplitude has been linked to different autonomic markers, including higher cortisol levels (86), pain perception (87), myocardial functional response to stress (88), depression (43), and overload response in autonomic dysfunction (40). A similar rsHEP modulation has been observed in other disorders with maladaptive responses to environmental demands (89,90–92). As in other populations [patients with hypertension (19) and patients with borderline personality disorder (46,49)], the increased trait-like rsHEP in patients with bvFTD was consistent with the opposite pattern (more positive HEP amplitude) reported during active interoceptive-emotional tasks (15,17,69). These results can be understood in the twofold model of neurovisceral integration (22,93,94). In this model, neurovisceral responses at resting (increased rsHEP) can be interpreted as basal interoceptive hyperactivation driven by allostatic-interoceptive overload. The reduced autonomic responsiveness during task (decreased HEP) can be understood as a lack of regulation triggered by exacerbated baseline modulations. These results suggest that a trait-like basal allostatic-interoceptive overload is accompanied by impaired neurovisceral responsiveness to external demands (22,93–95). Further combined assessment of resting state and active task HEP modulations would confirm this interpretation.

Right lateralization is consistent with previous HEP results (34,89,96), autonomic dysregulation (40), convergence of interoceptive and emotion processing (16), core emotional deficits in bvFTD (97), and AIN lateralization (1). In this sense, the scalp topography, time windows, amplitude modulation, and delimited results to bvFTD, together with previous evidence, suggest that rsHEP can index allostatic-interoceptive load (2–5).

Source space analyses linked rsHEP modulations to AIN regions (insula, anterior cingulate cortex, amygdala, hippocampus, supramarginal gyrus, superior temporal gyrus) in patients with bvFTD and HC subjects, consistent with other source location studies (20,55,56) and neuroanatomical models (2). These results, then, support the hypothesis of an allostatic-interoceptive overload in bvFTD. Compared with HC subjects, patients with bvFTD exhibited a hypoactivation of key interoceptive nodes (insula, anterior cingulate cortex, opercular region), consistent with interoceptive impairment of perception and control (34,89,98), along with hyperactivated areas linked to allostatic-interoceptive load in healthy aging (superior temporal pole, supramarginal gyrus) (57).

Furthermore, compared with AD, bvFTD had lower activation in interoceptive frontal areas. Conversely, hyperactivation in the inferior and medial temporal areas and in the inferior lingual, fusiform, and occipital cortices was observed in bvFTD. These regions are involved in allostatic visceral integration (somatosensory areas) and temporal regions in the AIN (1,99). Of particular relevance to our hypothesis, this pattern was specific for bvFTD.

Although sociocognitive alterations are affected in multiple neurodegenerative diseases (100,101), CS, EFs, and FER are canonically impaired in bvFTD (23,102–104). Particularly, current meta-analytic evidence (105,106) points to executive dysfunctions as core symptoms of bvFTD (107). More importantly, these impairments were selectively associated with enhanced rsHEP amplitudes only in patients with bvFTD. These deficits compromise everyday behavior and functionality of patients with bvFTD, increasing the maladaptive responses to environmental demands (103,104,108,109). These impairments could be the result of many different factors (110), including atrophy of different brain areas (111), impairment of other related cognitive processes (112), or different physiopathology mechanisms, such as stroke (113), vascular (114), neurodegeneration (115), metabolic disbalance (116), or neurotransmitter dysregulation (117). However, we found converging evidence that exacerbated rsHEP–increased modulation systematically predicted cognitive, executive, and emotional dysfunctions only in bvFTD. This association was not found in HC subjects or patients with AD. These cognitive functions in healthy populations and conditions other than bvFTD seem to be supported and modulated by allostatic-interoceptive load (1,16) and impaired by allostatic overload (9,18–22). In sum, these results suggest that allostatic-interoceptive overload in bvFTD is associated with the patients’ sociocognitive impairments.

In patients with bvFTD and HC subjects, anatomical correlates of rsHEP confirmed its grounding in the AIN (insula and anterior cingulate, with more right-lateralized involvement in bvFTD). Particularly, exacerbated rsHEP was related with increased AIN atrophy, critically compromised in bvFTD (118), and affected by allostatic-interoceptive load in healthy aging (119) and other disorders (120,121). This is in concordance with previous studies showing that patients with bvFTD have increased resting energy expenditure (a symptom of allostatic-interoceptive overload) compared with control and AD groups, associated with volume reductions in structures that are known to be involved in autonomic regulation (122).

FC analyses confirmed a selective association between rsHEP modulation and impaired right AIN in bvFTD. First, patients with bvFTD (but not AD) showed lower AIN connectivity compared with HC subjects. Second, the more negative the rsHEP in patients with bvFTD, the lower the AIN connectivity. Third, the specificity of AIN involvement in rsHEP-increased responses in bvFTD was confirmed by null associations between rsHEP and AIN connectivity in HC subjects and participants with AD as well as nonsignificant associations with other functional networks (SN, EN, DMN, VN, MN). Typically, bvFTD presents altered connectivity of the SN (123–125) but also the DMN (30), both partially involved with the AIN (1,27). Crucially, the SN has been associated with tracking of moment-to-moment body states (124) and implicated in the interaction between interoceptive awareness tracked by HEP and emotion recognition tasks (15,81). In addition, some hubs of the DMN are involved in allostatic-interoceptive load (1,126). However, previous results of SN and DMN in bvFTD are controversial, with reduced (125) or increased (27) DMN connectivity and SN abnormalities that seem absent in familial bvFTD (30) or unspecific to bvFTD given their presence in AD (127). These results provide novel support for a more extended and specific AIN disruption in bvFTD, selectively associated with ongoing exacerbated rsHEP.

The absence of association between SN or DMN with rsHEP modulations in patients with bvFTD, suggests that exacerbated rsHEP is specifically associated with reduced connectivity of certain hubs of the AIN in bvFTD and unrelated with other impairments in SN or DMN dysfunctions. In this sense, the AIN is involved in in a wide range of cognitive, executive, and emotional phenomena that can be explained by their reliance on allostatic-interoceptive load (1). To our knowledge, this is the first study evidencing that the moment-to-moment cortical processing of body signals in bvFTD is selectively and specifically associated with connectivity of the AIN.

Traditionally, distinctions between neurologic and psychiatric diseases have been overestimated (108,128,129). Currently, bvFTD provides a model combining neurologic and psychiatric approaches. The allostatic and interoceptive frameworks have been widely applied in psychiatry, but their use in neurology is more scarce and the transnosological implications are unexplored. Patients with bvFTD have been systematically characterized by a wide range of behavioral and personality changes, often hindering timely diagnosis and treatment (130). Allostatic-interoceptive overload have been more thoroughly assessed in psychiatric conditions (131,132). Connecting allostatic-interoceptive overload and interoceptive dysregulation with large-scale behavioral dysfunction can help to reduce the schism between psychiatry and neurology (108) while bringing more convergent clinical and biomarker insights (2,6). Thus, our work sets the basis for future studies applying an allostatic-interoceptive dimensional and transnosological framework across different brain diseases.

In this work, we show that exacerbated rsHEP in bvFTD is associated with different neuroanatomical markers of allostatic-interoceptive overload, including source space, anatomy, and specific AIN connectivity. These deficits also selectively explain the sociocognitive impairments in bvFTD. However, our work features some limitations that future work could address. First, it would be desirable to connect the rsHEP alterations in bvFTD with independent measures of allostasis and interoception, which have already reported in bvFTD separately. For instance, allostatic dysregulation via energy expenditure has been reported in bvFTD (122), and we have previously shown interoceptive deficits in bvFTD (17,74). Thus, future studies may use rsHEP alterations in bvFTD to test their impact in independent measures of allostasis and interoceptive performance and also evaluate their specificity of such disturbances in bvFTD relative to AD. Second, diagnoses were based on standardized clinical assessments in the absence of neuropathological confirmation. However, we have followed international standards (23,55,133) and multimodal studies with the current clinical criteria (17,55,60,134). Third, the patient samples had moderate sizes. Although other studies reported replicable findings with similar or smaller groups (17,135,136), our approach should be tested using larger samples. In addition, this caveat is counteracted by stringent control of clinical variables, systematic diagnostic procedures, and harmonized assessments. Similarly, the convergent cognitive, electrophysiological, neuroanatomical, and FC results, with moderate to large effect sizes, further attest to the robustness of the sample. Further longitudinal studies should evaluate the primary or secondary nature of allostatic-interoceptive overload in the neurodegenerative and clinical profile of patients with bvFTD.

## Conclusions

Multiple disparate deficits in bvFTD, including behavioral maladaptation (23), cognitive impairments (in CS, EFs, FER, and interoception) (17), autonomic disbalance (11), electrophysiological atypicalities (HEP), and atrophy and altered connectivity of frontoinsulotemporal hubs (15,17), could be better integrated into an allostatic-interoceptive overload framework. In such account, the interoceptive signals are parameterized to anticipate changing needs, evaluate priorities, and prepare the organism to satisfy those priorities before leading to errors (137,138). Physiological and behavioral adaptation to environmental demands relies on the integration of body state signals with socioemotional stimuli (15), situational context (12,13), and self-protection (139) processes, which are impaired in bvFTD. A novel predictive coding framework based on multimodal markers may provide fruitful insights on the physiological and behavioral underpinnings of the disease.

## Supplementary Material

Supplementary

## Figures and Tables

**Table 1.** Demographic and Neuropsychological Data

Variable	bvFTD, *n* = 19	HC, *n* = 42	AD, *n* = 33	Statistics, All Groups	Post Hoc Comparisons	
					Groups	*p* Value
Demographic Data						
Sex, female:male, *n*	5:14	23:19	20:13	χ^2^_2_ = 6.08 *p* = .04^a^	HC-bvFTD	.07^a^
					HC-AD	.78^a^
					bvFTD-AD	.03^a^
Age, years	68.57 (1.92)	69.87 (1.50)	74.65 (1.55)	*F*_2,91_ = 2.13 *p* = .08^b^	HC-bvFTD	.85^c^
					HC-AD	.07^c^
					bvFTD-AD	.04^c^
Education, years	14.57 (0.91)	13.64 (0.71)	11.20 (0.74)	*F*_2,91_ = 3.30 *p* = .01^b^	HC-bvFTD	.70^c^
					HC-AD	.06^c^
					bvFTD-AD	.03^c^
Neuropsychological Assessment						
Cognitive state (MoCA)	22.22 (0.92)	25.66 (0.75)	16.48 (0.72)	*F*_2,52_ = 16.12 *p* < .001^b^	HC-bvFTD	.01^c^
					HC-AD	<.001^c^
					bvFTD-AD	<.001^c^
Executive functions (IFS)	19.66 (0.90)	23.45 (0.78)	14.43 (0.71)	*F*_2,54_ = 36.99 *p* < .001^b^	HC-bvFTD	.01^c^
					HC-AD	<.001^c^
					bvFTD-AD	<.001^c^
Facial emotion recognition	10.16 (2.63)	12.35 (1.80)	9.78 (2.83)	*F*_2,53_ = 8.74 *p* < .001^b^	HC-bvFTD	.009^c^
					HC-AD	<.001^c^
					bvFTD-AD	.86

Data are presented as mean (SD), with the exception of sex.

AD, Alzheimer’s disease; bvFTD, behavioral variant frontotemporal dementia; HC, healthy control; IFS, Ineco Frontal Screening; MoCA, Montreal Cognitive Assessment.

a *p* Values calculated via χ^2^ test.

b *p* Values calculated via independent measures analysis of variance.

c *p* Values calculated via Tukey honestly significant difference test.

**Table 2.** Multivariate Multiple Regressions: bvFTD and AD Relative to HC

Variable	CS					EFs					FER				
	β	SE	95% CI	*p*	*R* ^2^	β	SE	95% CI	*p*	*R* ^2^	β	SE	95% CI	*p*	*R* ^2^
bvFTD	−1.65	1.60	−4.9 to 1.5	.3	0.5	−1.10	1.50	−0.48 to 0.19	.5	0.6	−1.35	0.66	−2.7 to −0.04	.04	0.38
AD	−9.81^a^	1.21^a^	−12 to −7.4^a^	<.001^a^		−957^a^	1.11^a^	−12 to −7.3^a^	<.001^a^		−2.31^a^	0.50^a^	−3.3 to −1.3^a^	<.001^a^	
rsHEP	−0.02	0.01	−0.05 to 0.01^a^	.2		−0.01	0.01	−0.04 to 0.01	.4		−0.01^a^	0.06	−0.02 to 0.00^a^	.04^a^	
rsHEP × bvFTD	0.04^a^	0.02^a^	0.01 to 0.1^a^	.05^a^		0.06^a^	0.02^a^	0.01 to 0.1^a^	.013^a^		0.03^a^	0.0^a^	0.01 to 0.05^a^	.01^a^	
rsHEP × AD	0.01	0.02	−0.03 to 0.05	.6		0.01	0.02	−0.02 to 0.04	.6		0.01	0.01	−0.01 to 0.02	.22	

AD, Alzheimer’s disease; bvFTD, behavioral variant frontotemporal dementia; CS, cognitive state; EF, executive function; FER, facial emotion recognition; HC, healthy control; rsHEP, resting-state heartbeat evoked potential.

a Significant differences.
