# Supplementary material for: Allostatic-Interoceptive Overload in Frontotemporal Dementia
Source: Biol Psychiatry. Author manuscript; Available in PMC 2024 Jun 18. (PMC11184918; doi:10.1016/j.biopsych.2022.02.955)
Supplement: Supplementary [file NIHMS1998262-supplement-Supplementary.pdf]

Allostatic Interoceptive Overload in Frontotemporal Dementia

Supplementary Information

Table of contents

1. Power estimation
2. Atrophy results
3. Hd-EEG methods: preprocessing steps and number of trials of the hdEEG samples
4. MRI data acquisition, preprocessing, and analysis
5. Supplementary Table 4 (Table S4)
6. Supplementary rsHEP modulations results
7. Supplementary rsHEP source localization results
  7.1. bvFTD activation results
  7.2 HC activation results
  7.3 AD activation results
  7.4. Activation differences between HC and bvFTD
  7.5. Activation differences between HC and AD
  7.6 Activation differences between bvFTD and AD
  7.7: Source split analysis results
8. bvFTD specific associations of rsHEP and multiple socio-cognitive measures
  8.1 Socio-cognitive split analysis results
  8.2 bvFTD associations of rsHEP and multiple socio-cognitive measures: bootstrapped models
9. SBM associations with rsHEP modulations
  9.1. SBM results for bvFTD patients
  9.2. SBM results for HCs
  9.3. SBM results for AD patients
  9.4 Split analysis of the associations between brain structure and rsHEP modulation
10. Functional connectivity supplementary analyses
  10.1. Functional connectivity split analysis:
  10.2. Bayes factors' analyses
11. Machine learning analysis
  11.1. Machine learning methods
  11.2. Machine learning results
12. Supplementary references

1. Power estimation

To determine the sample size required for our multivariate multiple linear regression analyses, we ran a power estimation on *R* with the pwr library. We established an alpha level of 0.05, an effect size of $\eta^2 = 0.15$, and a power of 0.8. The final sample size of our study (with the smallest group featuring 19 participants) reaches a power of .96.

2. Atrophy results

To establish patterns of structural brain alteration in each patient group, we assessed gray matter integrity via voxel-based morphometry (VBM) relative to controls. To do so, we estimated and used w-score maps of the normalized and smoothed DARTEL outputs in order to analyze the images of each center together and avoid a resonator effect in our results. We accounted for the global atrophy pattern via a *t*-test between groups, corrected by TIV and sex (SPM12). Statistical significance was set at $P < 0.001$ (FDR-corrected, extent threshold = 50 voxels). We found significant atrophy patterns for bvFTD and AD patients, but not for PD patients. Results are presented in Table S1.

Table S1. VBM atrophy of patients compared to HCs.

Atrophy	Cluster	Brain region	Peak		Coordinates		
			t	*P*	x	y	z
**bvFTD patients**	391	L Tri. Inferior Frontal	4.69	< 0.001	-34.5	21	25.5
	72	R Precuneus (BA31)	3.79	< 0.001	13.5	-61.5	57
	106	R Postcentral	3.74	< 0.001	51	-16.5	-21
	75	L Supramarginal	3.62	< 0.001	-60	-33	31.5
	150	R MiddleTemporal	3.61	< 0.001	43.5	4.5	-30
	59	L Posterior frontal	3.54	< 0.001	-34.5	6	40.5
**AD patients**	10954	R Amygdala	5.02	< 0.001	30	-3	-28.5
		R Hippocampus	4.97	< 0.001	27	-25.5	-7.5
	255	R Angular	3.98	< 0.001	34.5	-55.5	37.5
	574	R Inferior Parietal	3.47	< 0.001	37.5	-52.5	48
	209	L Superior Frontal	3.97	< 0.001	-24	28.5	31.5
	53	R Middle Temporal	3.73	< 0.001	39	-64.5	15
	237	R Precuneus	3.52	< 0.001	1.5	-55.5	46.5
	96	R Precuneus	3.49	< 0.001	10.5	-60	51
	150	R Superior Parietal	3.39	< 0.001	18	-55.5	49.5
	74	L Medial Superior Frontal	3.46	< 0.001	-7.5	55.5	9
	10954	R Amygdala	5.02	< 0.001	30	-3	-28.5
	51	R Hippocampus	4.97	< 0.001	27	-25.5	-7.5
	255	R Angular	3.98	< 0.001	34.5	-55.5	37.5

BvFTD: behavioral variant frontotemporal dementia; HC: healthy controls; AD: Alzheimer's disease patients. R: right. L: left.

3. Hd-EEG methods: preprocessing steps and number of trials of the hdEEG samples

We instructed participants not to think about anything in particular while remaining still, awake, and with eyes closed –which avoids artifacts and other noisy visual signals(1). Data was recorded via a Biosemi Active-two 128-channel system at a sampling rate of 1024 Hz and down-sampled offline 512 Hz. We corrected the hdEEG for eye movements, eyeblink artifacts, and heart with independent component analysis(2) and with a visual inspection protocol(3-11). We set analog filters at 0.03 and 100 Hz. We also applied a digital bandpass filter between 0.5 and 40 Hz offline to remove unwanted frequency components. The reference was set to linked mastoids for recording and re-referenced offline to the average of all of the rest electrodes. We replaced malfunctioning channels via statistically weighted spherical interpolation (based on neighboring sensors)(12). R-values from ECG signal were identified with pan Tompkins function on HEPLAB toolbox(13).

Spatiotemporal clustering

Spatiotemporal cluster analysys was repeated 5000 times, with recombination and randomized resampling of the subject-wise averages before each repetition using the Monte Carlo method(14). After each repetition, we retained the t-value of the largest cluster identified. The proportion of *t*-values obtained after randomization that were greater than the initially identified cluster-level *t*-value was used to calculate a nonparametric *P*-value for the cluster. This approach addresses the problem of multiple comparisons across the dimensions of the electrode, time, and space(15, 16). The reported latencies of HEPs in EEG vary significantly(17). In consequence, the cluster analysis was applied at the sensor level in the time window between 200 to 800 ms after the R-peak, to avoid cardiac artifacts(18, 19) and following HEP contamination(8, 20-22). In addition, we calculated the effect size for all comparisons following Cohen's method(23). Considering that bvFTD and AD groups were different in terms of age and years of education, we used a control-group-derived Z parameter (i.e., patients versus controls). For each bvFTD and AD patient, we calculated normalized Z-scores using the parameters (mean and *SD*) derived from the respective control group. Then, we compared Z-scores across the two groups through a 5000 point-by-point Monte Carlo permutation test with bootstrapping(24), with Benjamini and Hochberg false discovery rate (FDR) correction(25). To ensure that different sample sizes did not drive the results in the AD and HC groups relative to bvFTD patients, we repeated all analyses on subsamples of AD and HCs and the full sample of bvFTD (all groups with equal numbers of subjects), with equal numbers of subjects, selected with a randomized procedure (randperm function).

Source localization

The PCD maps were obtained using a head model of three concentric spheres, in a predefined source space of 21,926 voxels (voxel size of 4 x 4 x 4 mm^3) of the Montreal Neurological Institute average brain. A brain segmentation of 116 anatomic compartments was implemented using the automated anatomical labeling atlas (AAL atlas(26)). BMA images were subject-wise normalized (total average power over all images equal to unity) and baseline-corrected (relative to a window between 300 and 50 ms preceding the heartbeat). Then, we selected the inverse solutions corresponding to the time window at which rsHEP modulations significantly differed between groups in the cluster analysis (see hdEEG results section for details). The mean activation map of each group was obtained for each hdEEG scalp distribution. We computed 161 activation maps (161 time points, sampling the time interval between 270 and 584 ms following the peak of the R wave of the heartbeat). For each set of images (obtained at a particular time point), the mean PCDs computed in each voxel were compared between group pairs via two-tailed non-parametric permutation tests (α = .05; 20000 randomizations). Results were corrected for multiple comparisons through the Benjamin and Hochberg FDR method(25). For statistical analyses, we used in-house MATLAB codes (MathWorks, USA). BMA images were visualized using the software Neuronic Tomography Viewer. To control for sample sizes differences across groups,

we repeated all analyses on subsamples of AD and HCs and the full sample of bvFTD (all groups with equal numbers of subjects), selected with a randomized.

Table S2. Number of trials of the hdEEG subsamples.

Variable	bvFTD	HC	AD	Statistics (all groups)
Number of trials	198 (30)	184 (22)	162 (22)	$F(2,86) = 0.52$ $P = .60$

Data presented as mean (*SD*). BvFTD: behavioral variant frontotemporal dementia; HCs: healthy controls; AD: Alzheimer's disease.

4. MRI data acquisition, preprocessing, and analysis

Center 1: Using a 3-T Phillips scanner with a standard head coil, we acquired whole-brain T1-rapid anatomical 3D gradient echo volumes, parallel to the plane connecting the anterior and posterior commissures, with the following parameters: repetition time (TR) = 8300 ms; echo time (TE) = 3800 ms; flip angle = 8º; 160 slices, matrix dimension = 224 x 224 x 160; voxel size = 1 mm x 1 mm x 1 mm. Also, functional spin echo volumes, parallel to the anterior-posterior commissures, covering the whole brain, were sequentially and ascendingly acquired with the following parameters: TR = 2640 ms; TE = 30 ms; flip angle = 90º; 49 slices, matrix dimension = 80 x 80 x 49; voxel size in plane = 3 mm x 3 mm x 3 mm; slice thickness = 3 mm; sequence duration = 10 minutes; number of volumes = 220.

Center 2: Using a 3-T Siemens Skyra scanner with a standard head coil, we acquired whole-brain T1-rapid gradient ecAo volumes, parallel to the plane connecting the anterior and posterior commissures, with the following parameters: repetition time (TR) = 2400 ms; echo time (TE) = 2000 ms; flip angle = 8º; 192 slices, matrix dimension = 256 x 256 x 192; voxel size = 1 mm x 1 mm x 1 mm. Finally, functional EP2D-BOLD pulse sequences, parallel to the anterior-posterior commissures, covering the whole brain, were acquired sequentially intercalating pair-ascending first with the following parameters fMRI parameters: TR = 2660 ms; TE = 30 ms; flip angle = 90º; 46 slices, matrix dimension = 76 x 76 x 46; voxel size in plane = 3 mm x 3 mm x 3 mm; slice thickness = 3 mm; sequence duration = 10.5 minutes; number of volumes = 240.

Surface-based morphometry (SBM) pre-processing and analysis

We processed all T1 images via SBM on the FreeSurfer software suite (v 6.0 https://surfer.nmr.mgh.harvard.edu/). SBM metrics included sensitive measures of atrophy (volume and thickness). SBM offers reliable region-specific metrics to analyze structural(27) and functional connectivity(28) changes by overcoming registration errors and therefore improving the parcellation process. Preprocessing included removal of nonbrain tissue, an automatic Talairach transformation, segmentation of the subcortical white matter (WM) and deep GM volumetric structures (including the hippocampus, amygdala, caudate, putamen, and ventricles), intensity normalization, tessellation of the GM-WM boundary, an automatic topology correction, and surface deformation following

intensity gradients to optimally place the GM/WM and GM/CSF borders at the location where the greatest shift in intensity defines the transition to the other tissue class(29). Full details on the implemented methods can be found in a previous report(29). Finally, we quantified the volume and thickness from each segmentation of the Desikan-Killiany atlas(30). To control the variability of images acquired with different scanners, the volume of both HC and patients was z-scored based on the mean and SD of the corresponding center's HC(31-33). The plain-text output of the FreeSurfer's pipeline was post-processed on Python (v 3.7.4, Python Software Foundation) and restructured for statistical analysis. To avoid potential biases stemming from differences among the participant's head size(34), we normalized the volume measures of each area relative to the estimated total intracranial volume (eTIV, also provided in FreeSurfer's results).

To test our predictions, we assessed the association of the rsHEP modulation with the cortical volume and thickness measures from whole-brain parcellation through Kendall correlations with Pingouin statistics packages (v 0.3.6)(35) on Python (v 3.7.4, Python Software Foundation) (Figure 1B). Statistical significance was set at $P < .05$, FDR-corrected. Again, we repeated all analyses on subsamples of AD and HCs and the full sample of bvFTD (all groups with equal numbers of subjects), selected with a randomized procedure.

Preprocessing of fMRI data for rsFC analysis

First, to ensure that magnetization achieved a steady-state, we discarded the first five volumes of each subject's resting-state recording. Images were then preprocessed using the Data Processing Assistant for Resting-State fMRI (DPARSF V2.3)(36), an open-access toolbox that generates an automatic pipeline for fMRI analysis. DPARFS works by calling the SPM12 and the Resting-State fMRI Data Analysis Toolkit (REST V.1.7). As in previous studies(6, 8), preprocessing steps included slice-timing correction (using the middle slice of each volume as the reference scan) and realignment to the first scan of the session to correct head movement (SPM functions)(21, 37-42). We regressed out six motion parameters, cerebrospinal fluid (CSF) and white matter (WM) signals to reduce potential effects of movement-related, physiological, and cardio-respiratory effects (REST v.1.7 toolboxes). Motion parameters were estimated during realignment, and CFS and WM masks were derived from the tissue segmentation of each subject's T1 scan in native space with SPM12 (after co-registration of each subject's structural image with the functional image). None of the participants showed head movements greater than 3 mm and/or rotations higher than 3°(43) and no differences in head motion among groups were found (Supplementary Table 2). Finally, images were normalized to the MNI space using the echo-planar imaging (EPI) template from SPM12(44), smoothed using an 8-mm full-width-at-half-maximum isotropic Gaussian kernel, and bandpass filtered between 0.01–0.1 Hz to correct and remove low-frequency drifts from the MRI scanner. Finally, we excluded recordings with movements greater than 3 mm and/or rotation movements higher than 3º(43). Motion parameters did not differ significantly among groups (Table S2)

Analysis of rsFC fMRI data

Based on the fMRI resting-state recordings, we evaluated associations between the rsHEP modulations and FC patterns. Images were pre-processed on the Data Processing

Assistant for Resting-State fMRI (DPARSF v 2.3)(45), an open-access toolbox which generates an automatic pipeline for fMRI analysis by calling SPM 12 and the Resting-State fMRI Data Analysis Toolkit (REST v.1.7). To control the variability of images acquired with different scanners, the connectivity measures of both HCs and patients were z-scored based on the mean and *SD* of the corresponding center's HCs(31-33).

Our hypothesis predicted that rsHEP modulations in bvFTD would correlate with AIN connectivity. Therefore, we implemented a seed analysis to examine associations between the HEP modulation and FC of the AIN(46) (Figure 1B). To test the specificity of our predictions for these networks, we also examined associations of the rsHEP modulation with the connectivity of five additional functional networks: the SN, the executive network (EN), the DMN, the visual network (VN), and the motor network (MN). By testing the specificity of rsHEP as a marker of interoceptive allostatic overload in bvFTD, we expected non-significant associations between rsHEP modulations and the control networks. In order to compare with relevant works in neurodegenerative diseases, we planted seeds on cubic regions of interest (ROIs) with a size of 7x7x7 voxels(32, 47, 48) for each network. Each group of seeds was located on different coordinates to capture each network's connectivity. For the AIN, we selected three regions, used to estimate the unified allostasis/interoceptive system and the connections between cortical, amygdalar, visceromotor regions, and primary interoceptive regions(46). These regions were: the right dorsal middle insula (dmIns, MNI coordinates: 41, 2, 3 and -41, 2, 3), the right anterior midcingulate (aMCC, MNI coordinates: 9, 22, 33), and the right dorsal amygdala (dAmy, MNI coordinates: 27, 3, − 12)(46). Specific nodes, and their connectivity with the rest of the brain, were selected for the control networks, namely: the bilateral dorsal anterior cingulate (ACC) for the SN(49) (MNI coordinates 10, 34, 24 and -10, 34, 24), the right and left superior frontal gyri for the EN(32, 48, 50) (MNI coordinates 30, -2, 62 and -30,-2,62), the posterior cingulate (PCC) for the DMN(51) (MNI coordinates: 3,-54,27 and -3,-54,27); 5), the primary visual cortex for the VN(52), and the primary motor cortex (M1) for the MN(53) (MNI coordinates: 32,-30,68 and -32, 30,68). To calculate the networks, we employed a non-linear dynamic connectivity fluctuation analysis (DCFA)(32), which includes Dynamic Functional Connectivity (DFC)(54) and takes into account BOLD signal fluctuations. This analysis allows measuring the connectivity in the time domain, by segmenting the BOLD signal into non-overlapping time windows. DCFA proved to be robust against parameter and scanner acquisition heterogeneity. The symbolic weights capture transient brain activity that conventional methods may disregard as noise. Moreover, DFCA measure outperformed linear measures across in the discrimination and classification of patients with bvFTD and AD(55).

Then we used standard masks(56) to isolate the voxels typically involved in each resting-state network. Finally, data were spatially averaged across all included voxels to obtain a resting-state time series per network. The resulting connectivity maps were compared between group pairs with two-tailed non-parametric permutation tests (P = .05 FDR-corrected). Furthermore, connectivity maps in each group were correlated with the respective rsHEP modulations through the SPM12's multiple regression module (FDR-corrected P = *.05).* Once again, we repeated all analyses on subsamples of AD and HCs and the full sample of bvFTD selected with a randomized procedure.

5. Supplementary Table 3 (Table S3)

Table S3. Movement parameters for the fMRI subsamples.

Variable	BvFTD	HC	AD	Statistics (all groups)
Translation	.09<br>(.05)	.09<br>(.05)	.09<br>(.05)	$F = 1.04$<br>$P = 0.38$
Rotation	.06<br>(.03)	.04<br>(.05)	.06<br>(.03)	$F = .94$<br>$P = 0.42$

bvFTD: behavioral variant frontotemporal dementia; HC: healthy controls; AD: Alzheimer's disease.

6. Supplementary rsHEP modulations results

To ensure that the results were not driven by the more significant number of subjects in the AD and HC groups relative to bvFTD, we repeated the analyses selecting an equal number of subjects. The MATLAB's function randperm was used to determine the random samples. The resulted sample was demographically matched (Table S4). rsHEP comparisons between bvFTD and HC revealed a significant cluster over right centro-temporal regions in the 290-430 ms post R-peak period (t-sum= -50512.61, cluster-level P =.01, d = 0.62 corrected for multiple comparisons in space and time, Figure S1A), with its maximum t-value at 330 ms. Specifically, bvFTD patients presented increased negative amplitude than HC, meaning larger negative rsHEP modulation. In the normalized bvFTD vs AD subsample comparison, the rsHEP modulation difference was higher for bvFTD than for AD between 190 and 310 ms (P <.05, FDR-corrected, d =.50, Figure S1B).

Table S4. Subsamples' demographic data

	bvFTD<br>$N = 19$	HC<br>$N = 19$	AD<br>$N = 19$	Statistics<br>(all groups)
Sex<br>(F:M)	5:14	10:9	12:7	$\chi^2 = 3.61$<br>$P = .16$[a]
Years<br>of age	68.57<br>(1.92)	68.89<br>(7.48)	73.16<br>(6.11)	$F = 0.84$<br>$P = .43$[a]
Years of<br>education	14.57<br>(0.91)	14.77<br>(3.42)	12.9<br>(5.36)	$F = 1.26$<br>$P = .29$[a]

Data presented as mean (*SD*), with the exception of sex.
bvFTD: behavioral variant frontotemporal dementia; HC: healthy controls; AD: Alzheimer's disease.
[a] *P*-values calculated via independent measures ANOVA.
[b] *P*-values calculated via chi-squared test ($\chi 2$).

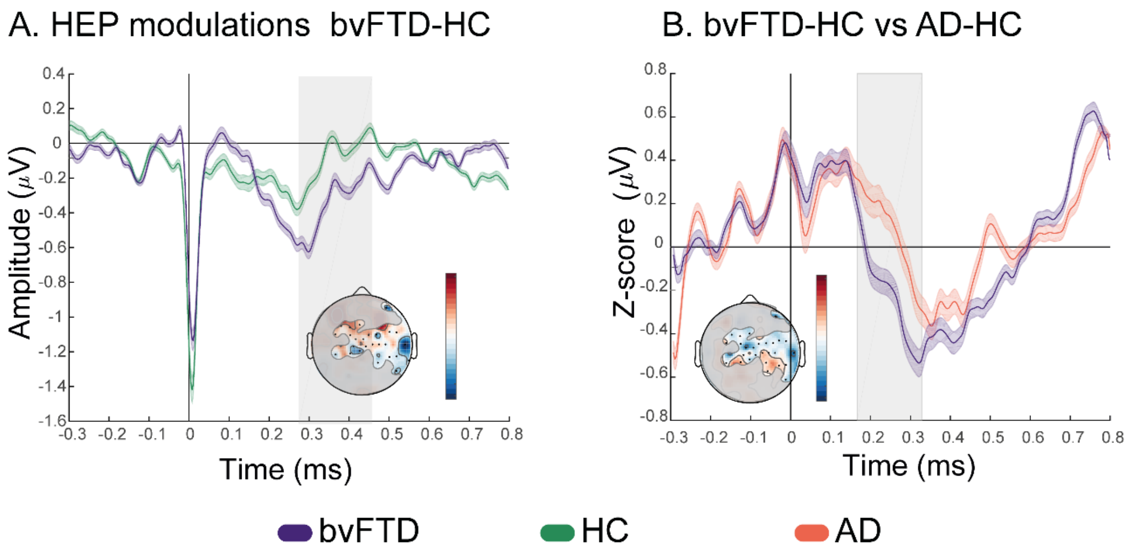

Figure S1: Split analysis results. **A.** rsHEP modulations during resting-state for the subsample of HC (green line) and bvFTD patients (violet line). Gray shaded boxes show statistically significant differences at $P = .05$ cluster corrected (from 290 to 430 ms). Scalp topographies show the significant electrodes of the cluster and the differences in amplitude (microvolts) between ERPs at 400 ms. **B.** Subtraction of the mean rsHEP modulations between bvFTD and HC (violet line), and AD and HC (pink line) in the cluster significant electrodes. Gray shaded boxes show statistically significant differences at $P = .05$ FDR-corrected (between 190 and 310 ms). Scalp topographies show the differences in amplitude (microvolts) at 250 ms and the electrodes used for the analysis.

7. Supplementary rsHEP source localization results

7.1. bvFTD activation results

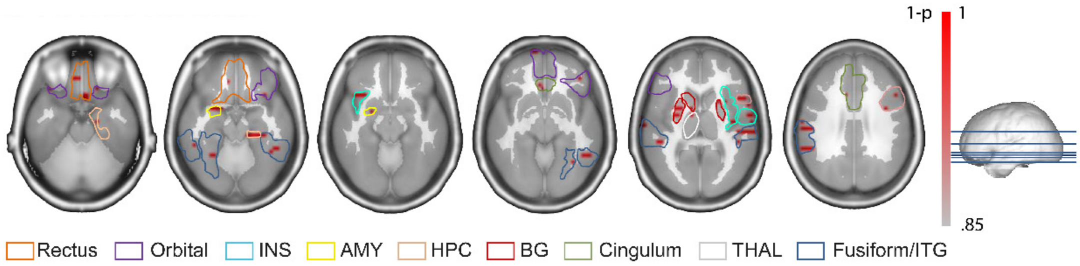

Figure S2: BMA images were visualized using the software Neuronic Tomography Viewer (Neuronic, Cuba) and segmented with the AAL atlas [12]. BvFTD: behavioral variant of frontotemporal dementia. INS: insula; AMY: amygdala; HPC: hippocampus; BG: basal ganglia; THAL: thalamus; ITG: inferior temporal gyrus.

Table S5. Voxels with maximum activations in patients with bvFTD.

Cortical region	MNI coordinates x	y	z		BA
Gyrus Rectus	6<br>-4	39<br>17	-24<br>-24	(right)<br>(left)	11
Inferior Frontal Gyrus (pars orbitalis)	37<br>-23	26<br>27	-24<br>-24	(right)<br>(left)	47
Middle Frontal Gyrus (orbital part)	-42	47	-4	(left)	10
Anterior Cingulate Cortex	6<br>8	30<br>20	-8<br>28	(right)<br>(left)	32
Inferior Frontal Gyrus (pars triangularis)	-42	26	4	(left)	45
Inferior Frontal Gyrus (pars opercularis)	44<br>-50	4<br>13	16<br>8	(right)<br>(left)	44
Insula	35<br>-39	17<br>-14	-12<br>8	(right)<br>(left)	13
Rolandic Operculum	-58	-5	8	(left)	48
Amigdala	23	2	-16	(right)	
ParaHippocampal Gyrus (PG)	25<br>-24	-6<br>-29	-32<br>-16	(right)<br>(left)	36
Caudate	9<br>-6	4<br>8	8<br>-4	(right)<br>(left)	
Putamen	26<br>-23	3<br>9	8<br>8	(right)<br>(left)	
Thalamus	15<br>-14	-24<br>-22	4<br>4	(right)<br>(left)	
Superior Temporal Gyrus	58<br>-18	-14<br>27	15<br>12	(right)<br>(left)	22
Inferior Temporal Gyrus	65<br>-53	-38<br>-26	-25<br>-20	(right)<br>(left)	20
Fusiform Gyrus	28<br>-32	-54<br>-61	-16<br>-8	(right)<br>(left)	37
Supramarginal Gyrus	58	-22	28	(right)	40

7.2 HC activation results

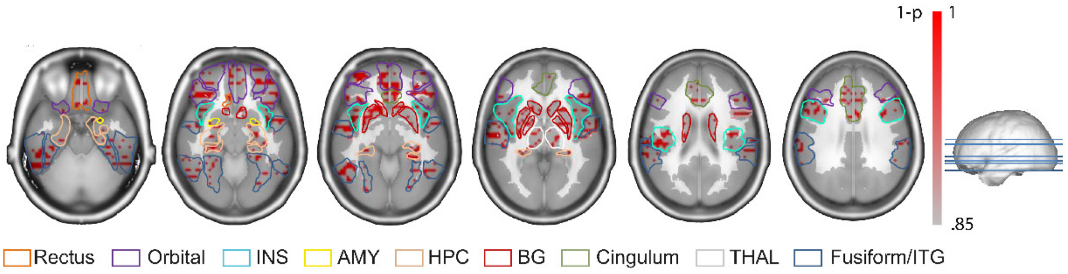

Figure S3: BMA images were visualized using the software Neuronic Tomography Viewer (Neuronic, Cuba) and segmented with the AAL atlas [12]. HC: healthy controls; INS: insula; AMY: amygdala; HPC: hippocampus; BG: basal ganglia; THAL: thalamus; ITG: inferior temporal gyrus.

Table S6. Voxels with maximum activations in HCs.

Cortical region	MNI coordinates				BA
	x	y	z		
Gyrus Rectus	4<br>-5	47<br>21	-24<br>24	(right)<br>(left)	11
Inferior Frontal Gyrus (pars orbitalis)	36<br>-41	32<br>48	-12<br>-12	(right)<br>(left)	47
Middle Frontal Gyrus (orbital part)	28<br>-33	55<br>42	-16<br>-8	(right)<br>(left)	10
Orbitofrontal Cortex	8<br>-6	55<br>42	-13<br>-8	(right)<br>(left)	10<br>11
Anterior Cingulate Gyrus	8<br>-7	42<br>35	4<br>24	(right)<br>(left)	32
Inferior Frontal Gyrus (pars triangularis)	48<br>-50	22<br>24	4<br>4	(right)<br>(left)	45
Inferior Frontal Gyrus (pars opercularis)	55<br>-54	12<br>10	28<br>20	(right)<br>(left)	44
Insula	43<br>-38	4<br>12	-8<br>4	(right)<br>(left)	13
Rolandic Operculum	46<br>-43	-25<br>-14	20<br>20	(right)<br>(left)	48
Amigdala	28<br>-26	2<br>-2	-15<br>-16	(right)<br>(left)	
Hippocampus (HPC)	27<br>-25	-18<br>-15	-12<br>-14	(right)<br>(left)	20
ParaHippocampal Gyrus (PG)	25<br>-29	-31<br>-15	-12<br>-14	(right)<br>(left)	37
Caudate	12<br>-11	17<br>19	-8<br>-8	(right)<br>(left)	
Putamen	22<br>-27	13<br>2	-8<br>4	(right)<br>(left)	
Pallidum	19<br>-18	-3<br>-2	0<br>0	(right)<br>(left)	
Thalamus	8<br>-13	25<br>-20	4<br>4	(right)<br>(left)	
Superior Temporal Gyrus	51<br>-59	-8<br>-20	-8<br>4	(right)<br>(left)	22
Inferior Temporal Gyrus	50<br>-53	-50<br>-33	-25<br>-20	(right)<br>(left)	20
Fusiform Gyrus	41<br>-40	-40<br>-33	-24<br>-20	(right)<br>(left)	37
Supramarginal Gyrus	63<br>-57	-32<br>-26	28<br>28	(right)<br>(left)	40

7.3 AD activation results

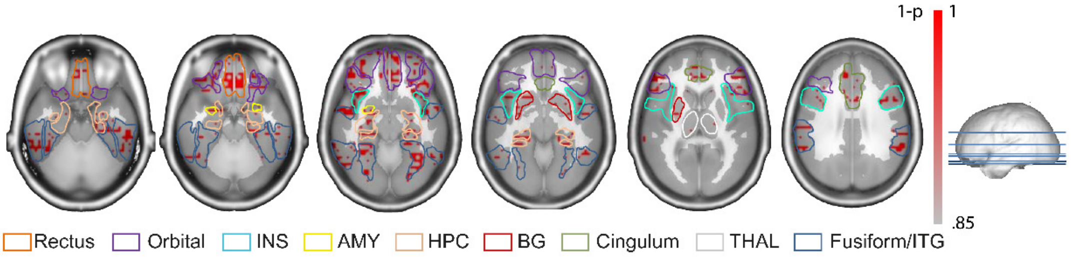

Figure S4: BMA images were visualized using the software Neuronic Tomography Viewer (Neuronic, Cuba) and segmented with the AAL atlas[57]. AD: Alzheimer's disease. INS: insula; AMY: amygdala; HPC: hippocampus; BG: basal ganglia; THAL: thalamus; ITG: inferior temporal gyrus.

Table S7. Voxels with maximum activations in patients with AD.

Cortical region	MNI coordinates x	y	z	Hemisphere	BA
Gyrus Rectus	5<br>-7	32<br>30	-20<br>20	Right<br>Left	11
Inferior Frontal Gyrus (pars orbitalis)	35<br>-31	35<br>26	-20<br>-20	Right<br>Left	47
Middle Frontal Gyrus (orbital part)	39<br>-31	56<br>45	-4<br>-10	Right<br>Left	10
Orbitofrontal Cortex	6<br>-10	53<br>37	-14<br>-12	Right<br>Left	11
Anterior Cingulate Gyrus	12<br>-5	40<br>41	12<br>8	Right<br>Left	32
Inferior Frontal Gyrus (pars triangularis)	49<br>-50	24<br>31	4<br>4	Right<br>Left	45
Inferior Frontal Gyrus (pars opercularis)	45<br>-50	14<br>11	4<br>4	Right<br>Left	44
Insula	43<br>-40	-1<br>5	4<br>-8	Right<br>Left	13
Rolandic Operculum	55<br>-56	-6<br>5	12<br>4	Right<br>Left	48
Amigdala	25<br>-25	-4<br>1	-14<br>20	Right<br>Left	
Hippocampus (HPC)	31<br>-30	-24<br>-19	-12<br>-14	Right<br>Left	20
ParaHippocampal Gyrus (PG)	27<br>-26	-39<br>-40	-8<br>-8	Right<br>Left	37
Caudate	14<br>-12	10<br>13	12<br>12	Right<br>Left	
Putamen	23<br>-22	11<br>3	-8<br>4	Right<br>Left	
Pallidum	19<br>-16	0<br>0	0<br>0	Right<br>Left	
Thalamus	9<br>-14	-24<br>-22	4<br>4	Right<br>Left	
Superior Temporal Gyrus	58<br>-54	-2<br>-1	4<br>-8	Right<br>Left	22

Inferior Temporal Gyrus	51<br>-49	-22<br>-13	-24<br>-28	Right<br>Left	20
Fusiform Gyrus	38<br>-32	-33<br>-41	-24<br>-20	Right<br>Left	37
Supramarginal Gyrus	59<br>-60	-39<br>-33	28<br>28	Right<br>Left	40

7.4. Activation differences between HC and bvFTD

Table S8. Voxels with maximum difference activation between HCs and bvFTD patients.

Cortical region	MNI coordinates			Hemisphere	BA
	x	y	z		
Gyrus Rectus	8<br>-6	38<br>18	-24<br>24	Right<br>Left	11
Inferior Frontal Gyrus (pars orbitalis)	38<br>-40	24<br>30	-16<br>-12	Right<br>Left	47
Middle Frontal Gyrus (orbital part)	27<br>-31	58<br>54	-16<br>-12	Right<br>Left	10
Orbitofrontal Cortex	5<br>-7	38<br>42	-12<br>-12	Right<br>Left	11
Anterior Cingulate Gyrus	7<br>-6	35<br>35	20<br>-8	Right<br>Left	32
Inferior Frontal Gyrus (pars triangularis)	54<br>-49	42<br>46	8<br>8	Right<br>Left	45
Inferior Frontal Gyrus (pars opercularis)	56<br>-51	18<br>13	8<br>8	Right<br>Left	44
Insula	34<br>-39	18<br>14	-11<br>-4	Right<br>Left	13
Rolandic Operculum	46<br>-53	-20<br>-6	20<br>8	Right<br>Left	48
Amigdala	23<br>-26	2<br>2	-16<br>-21	Right<br>Left	
Hippocampus (HPC)	25<br>-30	-14<br>-18	-20<br>-12	Right<br>Left	20
ParaHippocampal Gyrus (PG)	28<br>-24	-34<br>-30	-12<br>-16	Right<br>Left	37
Caudate	14<br>-12	10<br>18	16<br>-8	Right<br>Left	
Putamen	28<br>-21	3<br>13	8<br>-4	Right<br>Left	
Pallidum	-15	2	-4	Left	
Thalamus	14<br>-12	-15<br>10	16<br>4	Right<br>Left	
Superior Temporal Gyrus	57<br>-54	-6<br>-26	-4<br>4	Right<br>Left	22
Inferior Temporal Gyrus	53<br>-53	-31<br>-34	-24<br>-24	Right<br>Left	20
Fusiform Gyrus	37<br>-32	-30<br>-34	-24<br>-24	Right<br>Left	37
Supramarginal Gyrus	56<br>-57	-44<br>-28	28<br>32	Right<br>Left	40

7.5. Activation differences between HC and AD

Table S9. Voxels with maximum difference activation between HCs and AD patients.

Cortical region	MNI coordinates			Hemisphere	BA
	x	y	z		
Gyrus Rectus	8<br>-6	38<br>18	-24<br>24	Right<br>Left	11
Inferior Frontal Gyrus (pars orbitalis)	38<br>-40	24<br>30	-16<br>-12	Right<br>Left	47
Middle Frontal Gyrus (orbital part)	27<br>-31	58<br>54	-16<br>-12	Right<br>Left	10
Orbitofrontal Cortex	5<br>-7	38<br>42	-12<br>-12	Right<br>Left	11
Anterior Cingulate Gyrus	7<br>-6	35<br>35	20<br>-8	Right<br>Left	32
Inferior Frontal Gyrus (pars triangularis)	54<br>-49	42<br>46	8<br>8	Right<br>Left	45
Inferior Frontal Gyrus (pars opercularis)	56<br>-51	18<br>13	8<br>8	Right<br>Left	44
Insula	34<br>-39	18<br>14	-11<br>-4	Right<br>Left	13
Rolandic Operculum	46<br>-53	-20<br>-6	20<br>8	Right<br>Left	48
Amigdala	23<br>-26	2<br>2	-16<br>-21	Right<br>Left	
Hippocampus (HPC)	25<br>-30	-14<br>-18	-20<br>-12	Right<br>Left	20
ParaHippocampal Gyrus (PG)	28<br>-24	-34<br>-30	-12<br>-16	Right<br>Left	37
Caudate	14<br>-12	10<br>18	16<br>-8	Right<br>Left	
Putamen	28<br>-21	3<br>13	8<br>-4	Right<br>Left	
Pallidum	-15	2	-4	Left	
Thalamus	14<br>-12	-15<br>10	16<br>4	Right<br>Left	
Superior Temporal Gyrus	57<br>-54	-6<br>-26	-4<br>4	Right<br>Left	22
Inferior Temporal Gyrus	53<br>-53	-31<br>-34	-24<br>-24	Right<br>Left	20
Fusiform Gyrus	37<br>-32	-30<br>-34	-24<br>-24	Right<br>Left	37
Supramarginal Gyrus	56<br>-57	-44<br>-28	28<br>32	Right<br>Left	40

7.6 Activation differences between bvFTD and AD

Table S10. Voxels with maximum difference activation between AD and bvFTD

Cortical region	MNI coordinates x	y	z		BA
Anterior Pre-frontal cortex	5	52	20	(right)	10
	-5	18	20	(left)	
Anterior Cingulate Gyrus	1	45	20	(right)	32
	-5	23	20	(left)	
*Middle Temporal Gyrus	64	-34	-12	(right)	21
	-59	-8	-12	(left)	
*Inferior Temporal Gyrus	60	-39	-16	(right)	37
	-56	-54	-16	(left)	
*Fusiform Gyrus	28	-7	-40	(right)	37
	-39	-82	-16	(left)	
*Inferior Occipital Gyrus	40	-82	16	(right)	18
	-40	-83	-12	(left)	
*Ligual Gyrus	29	-91	-16	(right)	18
	-35	-87	-16	(left)	

Note: Areas at which activation in bvFTD patients was increased in comparison with that of Alzheimer's patients are denoted with an asterisk (*).

7.7. Source split analysis results

Table S11. Voxels with maximum difference activation between HC and bvFTD

Cortical region	MNI coordinates x	y	z		BA
Gyrus Rectus	4	52	-16	(right)	11
	-4	54	-16	(left)	
Inferior Frontal Gyrus (pars orbitalis)	45	45	-8	(right)	47
	-54	37	-4	(left)	
Middle Frontal Gyrus (orbital part)	5	50	-4	(right)	10
	-6	56	-8	(left)	
Orbitofrontal Cortex	39	55	4	(right)	11
	-28	59	4	(left)	
Anterior Cingulate Gyrus	5	47	20	(right)	32
	-5	45	16	(left)	
Inferior Frontal Gyrus (pars triangularis)	52	36	4	(right)	45
	-55	33	8	(left)	
Inferior Frontal Gyrus (pars opercularis)	54	14	20	(right)	44
	-61	16	20	(left)	
Insula	45	18	-4	(right)	13
	-32	20	-8	(left)	
Rolandic Operculum	59	7	12	(right)	48
	-54	2	12	(left)	
Amigdala	27	1	-16	(right)	
	-24	-3	-12	(left)	
Hippocampus (HPC)	29	-34	-4	(right)	37
	-30	-18	-12	(left)	
ParaHippocampal Gyrus (PG)	14	-15	-16	(right)	37
	-22	-4	-16	(left)	
Caudate	14	10	16	(right)	
	-12	18	-8	(left)	
Putamen	26	-8	8	(right)	

	-21	17	-8	(left)	
Putamen	16	1	-4	(right)	
	14	1	-4	(left)	
Thalamus	16	-30	8	(right)	
	-11	18	12	(left)	
Supplementary motor area	4	10	52	(right)	32
	-4	8	52	(left)	
* Superior Temporal Gyrus	58	-38	16	(right)	22
	-55	-12	4	(left)	
* Inferior Temporal Gyrus	38	-6	-54	(right)	20
	-45	-10	-40	(left)	
* Fusiform Gyrus	39	-11	-40	(right)	37
	-38	-11	-40	(left)	
* Supramarginal Gyrus	56	-36	24	(right)	40
	-64	-26	32	(left)	
* Precuneus	-	-	-	(right)	5
	-15	-53	68	(left)	

Table S12. Voxels with maximum difference activation between HC and AD

Cortical region	MNI coordinates x	y	z		BA
Gyrus Rectus	6	38	-28	(right)	11
	-3	20	-20	(left)	
Inferior Frontal Gyrus (pars orbitalis)	43	48	-4	(right)	46
	-51	41	-4	(left)	
Middle Frontal Gyrus (orbital part)	27	59	-12	(right)	11
	-23	61	-12	(left)	
Orbitofrontal Cortex	26	59	-8	(right)	11
	-33	58	-8	(left)	
Anterior Cingulate Gyrus	2	30	24	(right)	32
	-6	28	28	(left)	
Inferior Frontal Gyrus (pars triangularis)	47	40	12	(right)	45
	-45	47	8	(left)	
Inferior Frontal Gyrus (pars opercularis)	57	10	28	(right)	44
	-41	13	28	(left)	
Insula	29	15	-16	(right)	13
	-33	17	-16	(left)	
Rolandic Operculum	39	-24	20	(right)	48
	-41	-22	20	(left)	
Amigdala	25	2	-20	(right)	
	-29	2	-20	(left)	
Hippocampus (HPC)	27	-10	-16	(right)	20
	-27	-7	-20	(left)	
ParaHippocampal Gyrus (PGH)	26	-3	-32	(right)	36
	-23	-10	-32	(left)	
Caudate	12	17	8	(right)	
	-13	17	8	(left)	
Putamen	27	-6	8	(right)	
	-27	0	8	(left)	
Pallidum	28	-5	-4	(right)	
	-19	-5	-4	(left)	
Thalamus	6	-10	4	(right)	
	-14	-10	4	(left)	
Superior Temporal Gyrus	60	-23	8	(right)	22

	-53	-20	-8	(left)	
Middle Temporal Gyrus	61<br>-59	-25<br>-47	-4<br>20	(right)<br>(left)	21
Inferior Temporal Gyrus	52<br>-51	-60<br>-62	-8<br>-4	(right)<br>(left)	37
Fusiform Gyrus	40<br>-39	-57<br>-71	-16<br>-16	(right)<br>(left)	37
Supramarginal Gyrus	48<br>-57	-44<br>-50	24<br>24	(right)<br>(left)	40
Inferior Parietal Cortex	-<br>-35	-<br>-57	-<br>40	(right)<br>(left)	7
Angular Gyrus	-<br>36	-<br>-62	-<br>40	(right)<br>(left)	7
Cuneus Gyrus	5<br>-5	-70<br>-68	20<br>20	(right)<br>(left)	23
Inferior Occipital Gyrus	27<br>-	-85<br>-	16<br>-	(right)<br>(left)	18
Ligual Gyrus	22<br>-	-93<br>-	-8<br>-	(right)<br>(left)	18
Calcarine Gyrus	20<br>-	-96<br>-	-4<br>-	(right)<br>(left)	18

Table S13. Voxels with maximum difference activation between AD and bvFTD

Cortical region	MNI coordinates x	y	z		BA
Anterior Pre-frontal cortex	5<br>-4	58<br>56	4<br>0	(right)<br>(left)	10
Anterior Cingulate Gyrus	3<br>-4	51<br>46	8<br>8	(right)<br>(left)	32
Orbitofrontal Cortex	27<br>-6	61<br>61	-4<br>-6	(right)<br>(left)	11
Anterior prefrontal cortex	23<br>-31	61<br>60	8<br>-4	(right)<br>(left)	11
*Middle Temporal Gyrus	66<br>-63	-38<br>-50	8<br>12	(right)<br>(left)	21
*Inferior Temporal Gyrus	64<br>-53	-38<br>-54	-20<br>-20	(right)<br>(left)	37
*Fusiform Gyrus	38<br>-38	-72<br>-80	-16<br>-16	(right)<br>(left)	37
*Inferior Occipital Gyrus	29<br>-39	-89<br>-89	16<br>-12	(right)<br>(left)	18
*Ligual Gyrus	7<br>-8	-77<br>-76	0<br>0	(right)<br>(left)	18

Note: Areas at which activation in bvTD patients was increased in comparison with that of healthy controls or AD are denoted with an asterisk (*).

8. bvFTD specific associations of rsHEP and multiple socio-cognitive measures

Cognitive state (CS)

The Montreal Cognitive Assessment (MoCA)(58) tracks CS decline in patients with neurodegenerative diseases(59-61). It evaluates attention, concentration, EFs, memory, language,

visoconstructional and visuospatial skills, conceptual thinking, calculations, and orientation. Its maximum score is 30, with higher scores indicating better performance.

Executive function (EF)

The INECO Frontal Screening (IFS) battery(62) is sensitive and specific to detect frontal-executive dysfunction in patients with neurodegenerative diseases(62-65). It includes eight subtests that tap three EFs: response inhibition and set shifting, working memory, and abstraction capacity. Its maximum score is 30, with higher scores indicating better performance.

Facial emotion recognition (FER)

We used a standard FER task in which participants must identify emotional expressions depicted in a series of photographs(66). Stimuli comprise 35 faces selected from the larger emotion face set developed by Ekman(67). Participants are instructed to associate each facial expression with one of six possible emotion labels (happiness, surprise, sadness, fear, disgust, anger) or a neutral expression. A score (max. 15) was calculated from the percentage of correct responses.

We compared the rsHEP and cognitive associations in tandems (bvFTD and HCs; and then AD and controls). First, for each subject, we calculated the rsHEP modulation as the area under the curve (AUC) based on the significant spatiotemporal cluster discriminating bvFTD patients from HCs (Figure 1B). Similarly, for AD and HCs, we considered the largest spatiotemporal cluster discriminating AD patients from HCs. Thus, larger rsHEP negative modulations were indexed by more negative AUC values. Each AUC was calculated via MATLAB's trapz function. The MMLR model included CS, EF, and FER scores (dependent variables); and the interaction between the rsHEP modulation (AUC) and group (dummy variable: bvFTD-HCs) as predictors. The same MMLR was conducted for AD patients and HCs. The significance level was defined as $P = .05$. By performing a MMLR model with an overall *F*-test first, the following univariate *F*-tests were protected against an inflation of the overall error rate(68). In this sense we only inspected the univariate test when multivariate analysis yielded significant effects. All the analyses were performed with the lme4 library on *R* (version 3.5.2). To control for sample sizes differences across groups, we repeated all analyses on subsamples of AD and HCs as detailed above.

We evaluated to which extent the rsHEP in bvFTD was related to cognitive deficits at individual basis. To this end, we ran a MMLR between the three neuropsychological variables (CS, EF, FER) and the rsHEP modulations. We expected that the rsHEP would explain the socio-cognitive deficits only in bvFTD. In the bvFTD-HC model, results revealed significant multivariate effects for the interaction term between rsHEP modulation (AUC of rsHEP) and Group [$F(3,43) = 3.40$, $P = .025$, $\eta 2 = 0.19$]. The rsHEP modulation significantly predicted the three neuropsychological scores in bvFTD patients [CS: $F(3,45) = 4.76$, $P = .005$; EF: $F(3,45) = 8.30$, $P < .001$, FER: $F(3,45) = 10.43$, $P < .001$, for full results see Table S14, Figure 3A] but not in HCs. The larger negative rsHEP modulation, the higher the deficit of bvFTD patients in multimodal behavioral performance including CS, EF and FER.

In the AD-HC MMLR model, although we found a significant multivariate main effect of Group [$F(3,49) = 35.81$, $P < .001$, $\eta 2 = 0.68$], no significant interaction between rsHEP modulation and Group [$F(3,49) = .76$, $P = .51$, $\eta 2 = 0.04$]. Results showed that AD patients were outperformed in the three neuropsychological tests by HCs, independently of the rsHEP modulation [CS: $F(3,51) = 28.40$, $P < .001$; EF: $F(3,51) = 41.22$, $P < .001$, FER: $F(3,45) = 12.92$, $P < .001$; for full results, see Table S14, Figure 3B]. Our results indicate that while behavioral performance in AD patients and HCs was not directly related to rsHEP amplitude, negative rsHEP modulation was associated with neuropsychological deficits in bvFTD at individual basis.

Table s14: Multivariate multiple regressions

**bvFTD and HC**															
	**CS**					**EF**					**FER**				
Predictors	β	SE	*CI* (95%)	*P*	R^2	β	SE	*CI* (95%)	*P*	R^2	β	SE	*CI* (95%)	*P*	R^2
Group	-0.20	0.18	-0.56 – 0.17	.27	.24	-0.14	0.17	-0.48 – 0.19	.39	.35	-0.35	0.16	-0.67 – -0.02	.03	.41
HEP	0.07	0.15	-0.24 – 0.39	.63		0.23	0.14	-0.05 – 0.52	.11		0.02	0.14	-0.25 – 0.30	.15	
**HEP*Group**	**0.33**	**0.15**	**0.01 – 0.065**	**.046**		**0.42**	**0.14**	**0.12 – 0.71**	**.006**		**0.38**	**0.14**	**0.10 – 0.66**	**.009**	
**AD and HC**															
**Group**	**-0.82**	**0.10**	**-1.02 – -0.62**	**<.001**	.62	**-0.84**	**0.09**	**-1.02 – -0.67**	**<.001**	0.70	**-0.63**	**0.12**	**-0.88 – -0.39**	**<.001**	0.43
HEP	-0.10	0.09	-0.29 – 0.085	.27		-0.08	0.08	-0.25 – 0.09	.33		-0.25	0.11	-0.48 – -0.01	.03	
HEP*Group	0.003	0.10	-0.19 – 0.19	.97		0.05	0.08	-0.11 – 0.22	.53		0.16	0.11	-0.07 – 0.40	.16	

HC: healthy controls; bvFTD: behavioral variant frontotemporal dementia; AD: Alzheimer's disease; CS: cognitive state; EF: executive functions; FER: facial emotion recognition; rsHEP: resting state heart evoked potential. Bold lines highlight significant differences.

8.1 Socio-cognitive split analysis results

To evaluate the relations between neurovisceral markers of allostatic load (rsHEP) and cognitive deficits in bvFTD, we ran a multivariate multiple linear regression (MMLR(69)) considering rsHEP modulations as predictor and the three neuropsychological variables (CS, EF, FER) as outcomes. We compared the rsHEP and cognitive associations in the three groups (bvFTD, AD, and HCs). The MMLR model included CS, EF, and FER scores (dependent variables); and the interaction between the rsHEP modulation (AUC) and group (dummy variable: bvFTD-HCs-AD) as predictors. All the analyses were performed with the lme4 library on *R* (version 3.5.2). The significance level was defined as *P* = .05. We first performed an overall *F*-test on the MMLR model, and only inspected the univariate test if the multivariate model was significant.

Table S15. Multivariate multiple regressions with AD and HC subsamples.

**BvFTD relative to HC**												
	**CS**				**EF**				**FER**			
	β	SE	*P*	R^2	β	SE	*P*	R^2	β	SE	*P*	R^2
bvFTD	-1.50	1.62	0.4	0.3	-0.72	0.01	0.6	0.3	-1.18	0.38	.1	.4
rsHEP	-0.02	0.01	0.1		-0.01	0.01	0.3		-0.01	0.007	.08	
rsHEP*bvFTD	**0.05**	**0.02**	**0.04**		**0.06**	**0.02**	**0.012**		**0.02**	**0.01**	**.02**	
**BvFTD and AD relative to HC**												
	β	SE	*P*	R^2	β	SE	*P*	R^2	β	SE	*P*	R^2
bvFTD	-1.5	1.60	0.4	0.6	-0.72	1.45	0.61	0.6	-1.19	0.73	.11	.32
AD	**-8.02**	**1.35**	**<0.001**		**-8.21**	**1.27**	**<0.001**		**-1.97**	**0.64**	**<0.001**	
rsHEP	-0.02	0.02	0.13		-0.01	0.01	0.25		**-0.01**	**0.007**	**.08**	
rsHEP*bvFTD	**0.05**	**0.02**	**0.03**		**0.06**	**0.02**	**0.008**		**0.03**	**0.01**	**.020**	
rsHEP*AD	0.04	0.02	0.06		0.03	0.02	0.1		0.02	0.01	0.072	

HC: healthy controls; bvFTD: behavioral variant frontotemporal dementia; AD: Alzheimer's disease; CS: cognitive state; EF: executive functions; FER: facial emotion recognition; rsHEP: resting state heart evoked potential. Bold lines highlight significant differences.

8.2 bvFTD associations of rsHEP and multiple socio-cognitive measures: bootstrapped models

Table S16. cognitive screening

	Estimate	Confidence interval	Significance
rsHEP	0.009	-0.03 - – 0.001	
AD	1.44	-12.34 - -7.162	***
bvFTD	1.44	-4.46 - 1.27	
rsHEP*AD	0.01	-0.024 - 0.05	
rHEP*bvFTD	0.023	0.005- 0.091	**

AD: Alzheimer's disease; bvFTD: behavioral variant Frontotemporal Disease; rsHEP: resting state Heartbeat evoked potential;*P < .05; **P < .01; ***P < .001

Table S17. Executive functions

	Estimate	Confident interval	Significance
rsHEP	0.006	-0.02 - 0.007	
AD	1.55	-12.52 - -6.65	***
bvFTD	1.93	-4.09 - 3.51	
rsHEP*AD	0.02	-0.02 - 0.04	
rHEP*bvFTD	0.025	0.02 - 0.12	**

AD: Alzheimer's disease; bvFTD: behavioral variant Frontotemporal Disease; rsHEP: resting state Heartbeat evoked potential;*P<.05; **P<.01; ***P<.001

Table S18. Facial emotion recognition

	Estimate	Confident interval	Significance
rsHEP	0.003	-0.02 - 0.004	*
AD	10.41	-3.95 - -1.54	***
bvFTD	0.78	-2.61 - 0.53	
rsHEP*AD	0.005	-0.0003 - 0.022	
rHEP*bvFTD	0.013	0.004 - 0.056	*

AD: Alzheimer's disease; bvFTD: behavioral variant Frontotemporal Disease; rsHEP: resting state Heartbeat evoked potential;*P<.05; **P<.01; ***P<.001

9. SBM associations with rsHEP modulations

9.1. SBM results for bvFTD patients

Table S19. Associations between SBM results and rsHEP modulations for bvFTD patients.

**Region**	**Hemisphere**	***r***	***P***
Precentral	Left	.54	<.001
Caudal middle frontal	Left	.40	.001
Entorhinal	Left	.16	.01
Entorhinal	Left	.15	.01
Paracentral	Right	.13	.01

Rostral anterior cingulate	Left	.13	.01
Superiorparietal	Right	.13	.02
Temporalpole	Right	.13	.02
Insula	Right	.12	.02
Temporalpole	Right	.12	.02
Medial orbitofrontal	Left	.11	.02
Postcentral	Right	.10	.03
Insula	Left	.09	.03

9.2. SBM results for HCs

Table S20. Associations between SBM results and rsHEP modulations for HCs.

**Region**	**Hemisphere**	***r***	***P***
Temporal pole	Right	.28	.002
Isthmus cingulate	Left	.27	.003
Middle temporal	Left	.20	.004
Pericalcarine	Left	.19	.006
Hippocampus	Right	.15	.01
Frontal pole	Left	.15	.01
Cuneus	Left	.14	.01
Superior frontal	Left	.14	.01
Insula	Right	.13	.01
Temporal pole	Left	.13	.01
Rostral middle frontal	Right	.12	.01
Supramarginal	Right	.12	.01
Amygdala	Left	.11	.02
Frontal pole	Right	.11	.02
Insula	Left	.11	.03
Medial orbitofrontal	Left	.11	.03
Postcentral	Left	.11	.03
Entorhinal	Left	.11	.03
Lingual	Left	.11	.03
Insula	Right	.11	.03
Superior temporal	Right	.11	.04
Pericalcarine	Right	.11	.04
Supramarginal	Right	.11	.04
Temporal pole	Left	.11	.04
Precentral	Left	.11	.04

9.3. SBM results for AD patients

Table S21. Associations between SBM results and rsHEP modulations for AD patients.

Region	Hemisphere	*r*	*P*
Temporal pole	Right	0.51	0.01
Supramarginal	Left	0.39	0.01
Rostral middle frontal	Right	0.31	0.02
Frontal pole	Right	0.29	0.03
Temporal pole	Right	0.28	0.03
Middle temporal	Right	0.24	0.04
Lingual	Right	0.24	0.04

9.4 Split analysis of the associations between brain structure and rsHEP modulation

Table S22. AD subsample

Region	*Hemisphere*	*P*	*r*
Transversetempora	Left	0.019	0.4
Temporalpole	Right	0.021	0.38
Medialorbitofrontal	Left	0.024	0.38
Rostralmiddlefrontal	Right	0.030	0.32
Vessel	Right	0.038	0.28
Lateralorbitofrontal	Left	0.038	0.28
Supramarginal	Left	0.038	0.28
Cuneus	Left	0.038	0.28
Frontalpole	Right	0.042	0.24
Inferiortemporal	Right	0.042	0.24
Rostralanteriorcingulate	right	0.047	0.23

Table S23. HCs subsample

Region	*Hemisphere*	P value	R score
Amygdala	Left	0.001	0.25
Rostralmiddlefrontal	Right	0.005	0.17
Postcentral	Left	0.005	0.16
Cuneus	Right	0.006	0.15
Hippocampus	Right	0.007	0.15
Amygdala	Right	0.008	0.15
Superiorfrontal	Left	0.011	0.12
Pericalcarine	Right	0.015	0.12
Frontalpole	Right	0.015	0.12
Insula	Left	0.017	0.12
Precentral	Left	0.019	0.12
Cuneus	Left	0.019	0.12
Pericalcarine	Left	0.023	0.11

Frontalpole	Left	0.026	0.11
Medialorbitofrontal	Left	0.028	0.11
Temporalpole	Left	0.029	0.11
Parsopercularis	Left	0.031	0.11
Lateraloccipital	Left	0.031	0.11
Insula	Right	0.031	0.11
Rostralmiddlefrontal	Left	0.031	0.11
Entorhinal	Left	0.035	0.11
Parsorbitalis	Left	0.035	0.09
Paracentral	Right	0.044	0.09
Parstriangularis	Left	0.044	0.09
Caudalanteriorcingulate	Right	0.047	0.09
Parstriangularis	Left	0.049	0.08
Superiortemporal	Right	0.049	0.08
Inferiortemporal	Left	0.049	0.08

10. Functional connectivity supplementary analyses

10.1. Functional connectivity split analysis

Table S24: connectivity matrix results

	**AIN**		**SN**		**EN**		**DMN**		**VN**		**MN**	
	r	***P***	***r***	***P***	***r***	***P***	***r***	***P***	***r***	***P***	***r***	***P***
**bvFTD**	0.42	0.03	0.37	0.05	0.19	0.06	0.12	0.07	0.17	0.06	0.13	0.07
**HC**	0.13	0.07	0.14	0.07	0.17	0.06	0.16	0.07	0.16	0.06	0.12	0.07
**AD**	0.18	0.06	0.21	0.05	0.13	0.07	0.32	0.04	0.14	0.07	0.13	0.07

10.2. Bayes factors' analyses

We computed Bayes factors in order to grade the decisiveness of the evidence for and against the presence of a correlation (70-74) between AIN connectivity and the rsHEP modulation. The Bayes factor compares the adequacy of two models. Here, the first model is the alternative hypothesis – H1 – that postulates the presence of a correlation between the AIN connectivity and the rsHEP modulation. The second model is the null hypothesis – H0 – representing the absence of correlation between the two measures. We used conventional cut-offs: a Bayes factor >3 was considered as substantial evidence for the H1; conversely, a Bayes factor <0.33 represents moderate evidence in favor of the H0; and anything in between is weak or anecdotal evidence(71). All analyses were performed with the *BayesFactor* library(75) in R. Only bvFTD patients showed moderate evidence (BF_{H1} = 8.96) for a positive correlation between AIN rsFC and the rsHEP modulation (see Table S21).

Table S25. Bayes factors' results

Group	BF_{H1}	Evidence Categorization
bvFTD	8.96	Moderate evidence for H1
HC	1.34	Anecdotal evidence for H0
AD	0.52	Moderate evidence for H0

11.0: Machine learning analysis

11.1. Machine learning methods

We used a multimodal machine learning approach to evaluate whether multimodal neuroanatomy of allostatic interoceptive regions, rather than other less specific neuroanatomical regions, would better discriminate bvFTD patients relative to AD and HCs. Following machine-learning analysis guidelines(76), we split the datasets in a ratio of 80% for training, and 20% for testing, using random division, to test for generalizability without employing the testing dataset during the validation phase for out of k-folds predictions. The 80/20 split is the gold-standard for obtaining robust cross-validation results across neuroimaging research(77). We trained the model with all anatomical data: rsHEP sources, volumetric data, and seeds of all rsFC networks. For the training phase in all our analyses, following best practices, we employed a k-fold cross-validation for Bayesian hyper-parameter tuning(78, 79). First, we ran a classifier to discriminate between bvFTD patients from HCs. Then, to test the specificity of potential results from that analysis, we examined the classification accuracy between bvFTD and AD patients. To establish which features were the most relevant for each classification scheme, we employed the feature importance analysis technique, building in our machine learning algorithm(80). We used a GBM classifier library called eXtreme Gradient Boosting (XGBoost)(80), because of its high accuracy and robustness relative to other algorithms, tuning its hyper-parameters by Bayesian Optimization(81, 82). GBMs are based on the gradient boosting technique, in which ensembles of decision trees iteratively attempt to correct the classification errors of their predecessors by minimizing a loss function (i.e., a function representing the difference between the estimated and true values) while pointing in the negative gradient direction(83). The XGBoost classifier provides both parallel computation tree boosting, enabling fast and accurate predictions which have proven successful in several fields(84); and also regularized boosting, helping to reduce overfitting and thus providing more generalizable results. Following guidelines for reporting machine learning results(85), classification accuracy values were accompanied by (i) calculations of the area under the curve (AUC) of the receiver operating characteristic (ROC) curve, and (ii) confusion matrices capturing the sensitivity and specificity of each classification.

11.2. Machine learning results

The machine learning classification between bvFTD patients and HCs, based on an XGBoost algorithm including all neuroanatomical measures (HEP source space, brain structure and rsFC networks), achieved the highest accuracy rate with top features indexing allostatic interoceptive regions. The classificatory relevance was highest for the AIN rsFC feature, followed by activity of the medial frontal gyrus. The area under the curve (AUC) of the receiver operating characteristic (ROC) was 0.89 (80% sensitivity, 88% specificity) shown in the confusion matrix (Figure S3A). The classification between AD patients and HCs, achieved less accuracy rate. The classificatory relevance was highest for the rsFC of the DMN, followed by the activity of the left middle temporal gyrus. The AUC was 0.83 (82% sensitivity, 80% specificity) (Figure S3B). The classification between bvFTD and AD patients achieved an 82% accuracy rate. The classificatory relevance was highest for the AIN rsFC and followed by the activity of the left middle frontal gyrus. The AUC was 0.81 (85% sensitivity, 78% specificity) (Figure S3C).

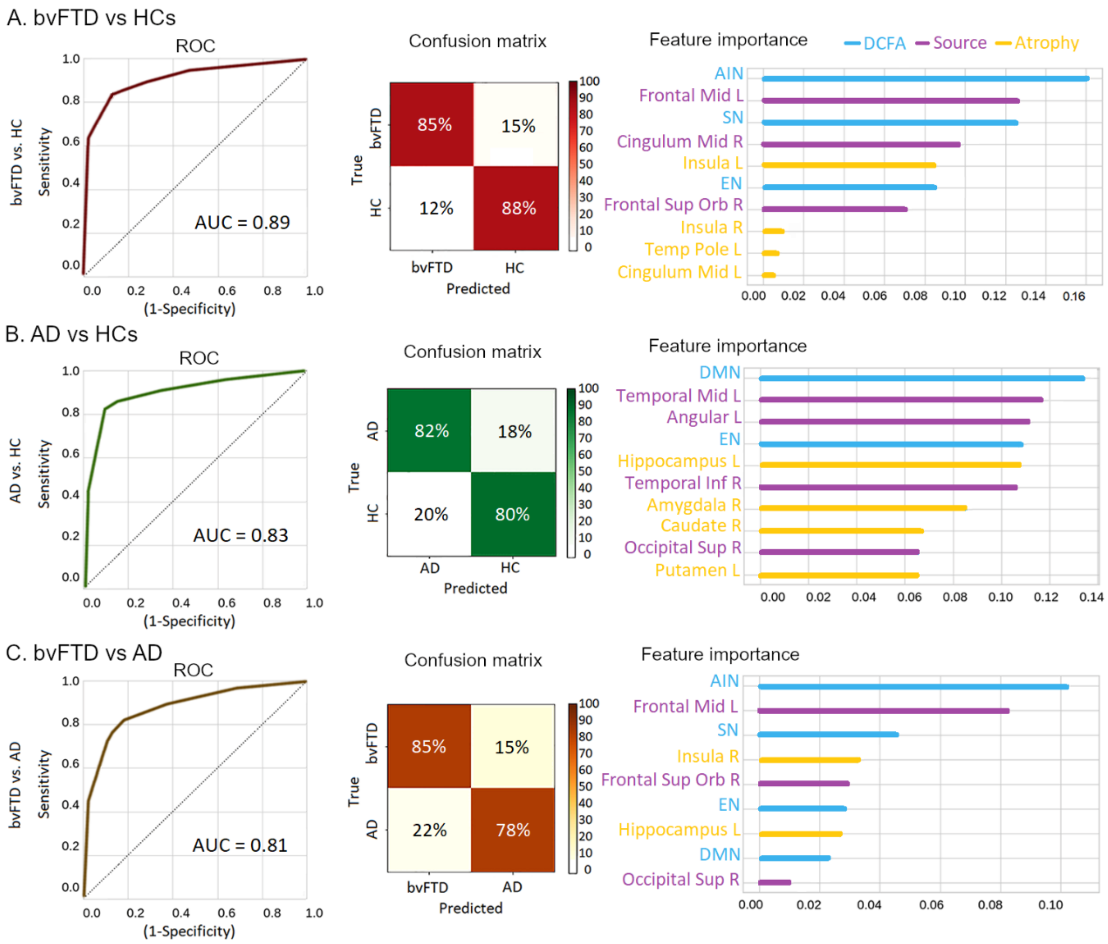

Figure S5: Machine learning results. (A) bvFTD patients vs controls. ROC curve indicating specificity (true positive rate) and sensitivity (false positive rate), while calculating the area under the curve. Confusion matrix for true label and predicted label accuracy details. Feature importance plot of the most relevant features for the classification. Results show an 87% accuracy rate, an AUC of 0.89, a sensitivity of 80%, and a specificity of 88%, with the functional connectivity of the AIN as the top feature, followed by the activation of the middle frontal gyrus, a key region of the allostatic processes. (B) AD patients vs HCs. Results show an 81% accuracy rate, with an AUC of 0.83, with a sensitivity of 82% and a specificity of 80% specificity. The functional connectivity of the DMN and the activity of the middle temporal gyrus were the top features. (C) bvFTD vs AD patients. Results yielded an 82% accuracy rate, an AUC of 0.81, a sensitivity of 85%, and a specificity of 78%, with the AIN and middle frontal gyrus as the top features. ROC: Receiver operating characteristic, AUC: Area under the curve, bvFTD: behavioral variant of frontotemporal dementia; AD: Alzheimer's disease; HCs: healthy controls; AIN: allostatic interoceptive network; SN: salience network; DMN: default mode network; EN: executive network.

12. Supplementary references

1. Dottori M, Sedeño L, Caro MM, Alifano F, Hesse E, Mikulan E, et al. (2017): Towards affordable biomarkers of frontotemporal dementia: A classification study via network's information sharing. *Scientific Reports*. 7:3822.
2. Kim D, Kim S-K (2012): Comparing patterns of component loadings: Principal Component Analysis (PCA) versus Independent Component Analysis (ICA) in analyzing multivariate non-normal data. *Behavior research methods*. 44:1239-1243.

3. Pollatos O, Schandry R (2004): Accuracy of heartbeat perception is reflected in the amplitude of the heartbeat-evoked brain potential. *Psychophysiology*. 41:476-482.
4. Terhaar J, Viola FC, Bar KJ, Debener S (2012): Heartbeat evoked potentials mirror altered body perception in depressed patients. *Clinical neurophysiology : official journal of the International Federation of Clinical Neurophysiology*. 123:1950-1957.
5. Schandry R, Montoya P (1996): Event-related brain potentials and the processing of cardiac activity. *Biological psychology*. 42:75-85.
6. Salamone PC, Esteves S, Sinay VJ, Garcia-Cordero I, Abrevaya S, Couto B, et al. (2018): Altered neural signatures of interoception in multiple sclerosis. *Hum Brain Mapp*. 39:4743-4754.
7. Garcia-Cordero I, Esteves S, Mikulan EP, Hesse E, Baglivo FH, Silva W, et al. (2017): Attention, in and Out: Scalp-Level and Intracranial EEG Correlates of Interoception and Exteroception. *Frontiers in neuroscience*. 11:411.
8. Yoris A, Abrevaya S, Esteves S, Salamone P, Lori N, Martorell M, et al. (2018): Multilevel convergence of interoceptive impairments in hypertension: New evidence of disrupted body-brain interactions. *Hum Brain Mapp*. 39:1563-1581.
9. Dirlich G, Vogl L, Plaschke M, Strian F (1997): Cardiac field effects on the EEG. *Electroencephalography and clinical neurophysiology*. 102:307-315.
10. Garcia-Cordero I, Sedeno L, de la Fuente L, Slachevsky A, Forno G, Klein F, et al. (2016): Feeling, learning from and being aware of inner states: interoceptive dimensions in neurodegeneration and stroke. *Philos Trans R Soc Lond B Biol Sci*. 371.
11. Yoris A, Garcia AM, Traiber L, Santamaria-Garcia H, Martorell M, Alifano F, et al. (2017): The inner world of overactive monitoring: neural markers of interoception in obsessive-compulsive disorder. *Psychological medicine*. 47:1957-1970.
12. Courellis HS, Iversen JR, Poizner H, Cauwenberghs G (2016): EEG channel interpolation using ellipsoid geodesic length. *2016 IEEE Biomedical Circuits and Systems Conference (BioCAS)*: IEEE, pp 540-543.
13. Sedghamiz HJCAatFESoM (2014): Matlab implementation of Pan Tompkins ECG QRS detector.
14. Manly B (2007): *Randomization, bootstrap, and monte carlo methods in biology*. Boca Raton, FL: Chapman & Hall.
15. Chennu S, Noreika V, Gueorguiev D, Blenkmann A, Kochen S, Ibanez A, et al. (2013): Expectation and Attention in Hierarchical Auditory Prediction. *J Neurosci*. 33:11194-11205.
16. Maris E, Oostenveld R (2007): Nonparametric statistical testing of EEG- and MEG-data. *Journal of neuroscience methods*. 164:177-190.
17. Kern M, Aertsen A, Schulze-Bonhage A, Ball T (2013): Heart cycle-related effects on event-related potentials, spectral power changes, and connectivity patterns in the human ECoG. *Neuroimage*. 81:178-190.
18. Kern M, Aertsen A, Schulze-Bonhage A, Ball TJN (2013): Heart cycle-related effects on event-related potentials, spectral power changes, and connectivity patterns in the human ECoG. 81:178-190.
19. Park HD, Blanke O (2019): Heartbeat-evoked cortical responses: Underlying mechanisms, functional roles, and methodological considerations. *Neuroimage*. 197:502-511.
20. Abrevaya S, Fittipaldi S, Garcia AM, Dottori M, Santamaria-Garcia H, Birba A, et al. (2020): At the Heart of Neurological Dimensionality: Cross-Nosological and Multimodal Cardiac Interoceptive Deficits. *Psychosomatic Medicine*. 82:850-861.
21. García-Cordero I, Sedeño L, De La Fuente L, Slachevsky A, Forno G, Klein F, et al. (2016): Feeling, learning from and being aware of inner states: interoceptive dimensions in neurodegeneration and stroke. *Philos T R Soc B*. 371:20160006.
22. Yoris A, García AM, Traiber L, Santamaría-García H, Martorell M, Alifano F, et al. (2017): The inner world of overactive monitoring: neural markers of interoception in obsessive-compulsive disorder. 47:1957-1970.
23. Oostenveld R, Fries P, Maris E, Schoffelen J-MJCi, neuroscience (2011): FieldTrip: open source software for advanced analysis of MEG, EEG, and invasive electrophysiological data. 2011.

24. Manly BF (2006): *Randomization, bootstrap and Monte Carlo methods in biology*. CRC press.
25. Benjamini Y, Hochberg YJJotRsssB (1995): Controlling the false discovery rate: a practical and powerful approach to multiple testing. 57:289-300.
26. Tzourio-Mazoyer N, Landeau B, Papathanassiou D, Crivello F, Etard O, Delcroix N, et al. (2002): Automated anatomical labeling of activations in SPM using a macroscopic anatomical parcellation of the MNI MRI single-subject brain. 15:273-289.
27. Clarkson MJ, Cardoso MJ, Ridgway GR, Modat M, Leung KK, Rohrer JD, et al. (2011): A comparison of voxel and surface based cortical thickness estimation methods. *NeuroImage*. 57:856-865.
28. Zuo X-N, Xu T, Jiang L, Yang Z, Cao X-Y, He Y, et al. (2013): Toward reliable characterization of functional homogeneity in the human brain: preprocessing, scan duration, imaging resolution and computational space. *NeuroImage*. 65:374-386.
29. Fischl B (2012): FreeSurfer. *NeuroImage*. 62:774-781.
30. Desikan RS, Segonne F, Fischl B, Quinn BT, Dickerson BC, Blacker D, et al. (2006): An automated labeling system for subdividing the human cerebral cortex on MRI scans into gyral based regions of interest. *NeuroImage*. 31:968-980.
31. Salamone PC, Legaz A, Sedeño L, Moguilner S, Fraile-Vazquez M, Campo CG, et al. (2021): Interoception primes emotional processing: Multimodal evidence from neurodegeneration. 41:4276-4292.
32. Moguilner S, García, A. M., Perl, Y. S., Tagliazucchi, E., Piguet, O., Kumfor, F., Reyes, P., Matallana, D., Sedeño, L., & Ibáñez, A. (2021): Dynamic brain fluctuations outperform connectivity measures and mirror pathophysiological profiles across dementia subtypes: A multicenter study. *NeuroImage*. 225.
33. Donnelly-Kehoe PA, Pascariello GO, García AM, Hodges JR, Miller B, Rosen H, et al. (2019): Robust automated computational approach for classifying frontotemporal neurodegeneration: Multimodal/multicenter neuroimaging. 11:588-598.
34. Whitwell JL, Crum WR, Watt HC, Fox NC (2001): Normalization of cerebral volumes by use of intracranial volume: implications for longitudinal quantitative MR imaging. *American Journal of Neuroradiology*. 22:1483-1489.
35. Vallat RJJoOSS (2018): Pingouin: statistics in Python. 3:1026.
36. Yan C, Zang YJFisn (2010): DPARSF: a MATLAB toolbox for" pipeline" data analysis of resting-state fMRI. 4:13.
37. Barttfeld P, Wicker B, Cukier S, Navarta S, Lew S, Leiguarda R, et al. (2012): State-dependent changes of connectivity patterns and functional brain network topology in autism spectrum disorder. *Neuropsychologia*. 50:3653-3662.
38. Barttfeld P, Wicker B, McAleer P, Belin P, Cojan Y, Graziano M, et al. (2013): Distinct patterns of functional brain connectivity correlate with objective performance and subjective beliefs. *Proceedings of the National Academy of Sciences*. 110:11577-11582.
39. García-Cordero I, Sedeño L, Fraiman D, Craiem D, de la Fuente LA, Salamone P, et al. (2015): Stroke and Neurodegeneration Induce Different Connectivity Aberrations in the Insula. *Stroke*. 46:2673-2677.
40. Sedeno L, Couto B, Garcia-Cordero I, Melloni M, Baez S, Morales Sepulveda JP, et al. (2016): Brain Network Organization and Social Executive Performance in Frontotemporal Dementia. *J Int Neuropsychol Soc*. 22:250-262.
41. Sedeño L, Couto B, Melloni M, Canales-Johnson A, Yoris A, Baez S, et al. (2014): How do you feel when you can't feel your body? Interoception, functional connectivity and emotional processing in depersonalization-derealization disorder. *PLoS One*. 9:e98769.
42. Melloni M, Billeke P, Baez S, Hesse E, de la Fuente L, Forno G, et al. (2016): Your perspective and my benefit: multiple lesion models of self-other integration strategies during social bargaining. *Brain*.
43. Supekar K, Menon V (2012): Developmental maturation of dynamic causal control signals in higher-order cognition: a neurocognitive network model. *PLoS computational biology*. 8:e1002374.

44. Ashburner J, Barnes G, Chen C-C, Daunizeau J, Flandin G, Friston K, et al. (2014): SPM12 manual. 2464.
45. Chao-Gan Y, Yu-Feng Z (2010): DPARSF: a MATLAB toolbox for "pipeline" data analysis of resting-state fMRI. *frontiers in Systems Neuroscience*. 4.
46. Kleckner IR, Zhang J, Touroutoglou A, Chanes L, Xia C, Simmons WK, et al. (2017): Evidence for a Large-Scale Brain System Supporting Allostasis and Interoception in Humans. *Nat Hum Behav*. 1.
47. Koslov K, Mendes WB, Pajtas PE, Pizzagalli DAJPS (2011): Asymmetry in resting intracortical activity as a buffer to social threat. 22:641-649.
48. Salamone PC, Legaz, A., Sedeño, L., Moguilner, S., Fraile-Vazquez, M., Campo, C. G., Fittipaldi, S., Yoris, A., Miranda, M., Birba, A., Galiani, A., Abrevaya, S., Neely, A., Caro, M. M., Alifano, F., Villagra, R., Anunziata, F., Oliveira, M. O., Pautassi, R. M., Slachevsky, A., … Ibañez, A. (2021): Interoception primes emotional processing: Multimodal evidence from neurodegeneration. . *The Journal of neuroscience : the official journal of the Society for Neuroscience, JN-RM-2578-20 Advance online publication*.
49. Seeley WW, Menon V, Schatzberg AF, Keller J, Glover GH, Kenna H, et al. (2007): Dissociable intrinsic connectivity networks for salience processing and executive control. 27:2349-2356.
50. Boord P, Madhyastha TM, Askren MK, Grabowski TJJNC (2017): Executive attention networks show altered relationship with default mode network in PD. 13:1-8.
51. Uddin LQ, Clare Kelly A, Biswal BB, Xavier Castellanos F, Milham MPJHbm (2009): Functional connectivity of default mode network components: correlation, anticorrelation, and causality. 30:625-637.
52. Saiote C, Tacchino A, Brichetto G, Roccatagliata L, Bommarito G, Cordano C, et al. (2016): Resting-state functional connectivity and motor imagery brain activation. 37:3847-3857.
53. Vahdat S, Darainy M, Milner TE, Ostry DJJJoN (2011): Functionally specific changes in resting-state sensorimotor networks after motor learning. 31:16907-16915.
54. Moguilner S, García, A. M., Mikulan, E., Hesse, E., García-Cordero, I., Melloni, M., Cervetto, S., Serrano, C., Herrera, E., Reyes, P., Matallana, D., Manes, F., Ibáñez, A., & Sedeño, L. (2018): Weighted Symbolic Dependence Metric (wSDM) for fMRI resting-state connectivity: A multicentric validation for frontotemporal dementia. *Scientific reports*. 8:11181.
55. Moguilner S, García AM, Mikulan E, Hesse E, García-Cordero I, Melloni M, et al. (2018): Weighted Symbolic Dependence Metric (wSDM) for fMRI resting-state connectivity: a multicentric validation for frontotemporal dementia. 8:1-15.
56. Shirer WR, Ryali S, Rykhlevskaia E, Menon V, Greicius MDJCc (2012): Decoding subject-driven cognitive states with whole-brain connectivity patterns. 22:158-165.
57. Tzourio-Mazoyer N, Landeau B, Papathanassiou D, Crivello F, Etard O, Delcroix N, et al. (2002): Automated anatomical labeling of activations in SPM using a macroscopic anatomical parcellation of the MNI MRI single-subject brain. *Neuroimage*. 15:273-289.
58. Nasreddine ZS, Phillips NA, Bédirian V, Charbonneau S, Whitehead V, Collin I, et al. (2005): The Montreal Cognitive Assessment, MoCA: a brief screening tool for mild cognitive impairment. *Journal of the American Geriatrics Society*. 53:695-699.
59. Santamaría-García H, Baez S, Reyes P, Santamaría-García JA, Santacruz-Escudero JM, Matallana D, et al. (2017): A lesion model of envy and Schadenfreude: legal, deservingness and moral dimensions as revealed by neurodegeneration. *Brain*. 140:3357-3377.
60. Damian AM, Jacobson SA, Hentz JG, Belden CM, Shill HA, Sabbagh MN, et al. (2011): The Montreal Cognitive Assessment and the mini-mental state examination as screening instruments for cognitive impairment: item analyses and threshold scores. *Dementia and geriatric cognitive disorders*. 31:126-131.
61. Roalf DR, Moberg PJ, Xie SX, Wolk DA, Moelter ST, Arnold SE (2013): Comparative accuracies of two common screening instruments for classification of Alzheimer's disease, mild cognitive impairment, and healthy aging. *Alzheimer's & Dementia*. 9:529-537.
62. Torralva T, Roca M, Gleichgerrcht E, Lopez P, Manes F (2010): INECO Frontal Screening (IFS): A brief, sensitive, and specific tool to assess executive functions in dementia–ERRATUM. *Journal of the International Neuropsychological Society*. 16:737-747.

63. Custodio N, Herrera-Perez E, Lira D, Roca M, Manes F, Báez S, et al. (2016): Evaluation of the INECO Frontal Screening and the Frontal Assessment Battery in Peruvian patients with Alzheimer's disease and behavioral variant Frontotemporal dementia. *ENeurologicalSci*. 5:25-29.
64. Ihnen J, Antivilo A, Muñoz-Neira C, Slachevsky A (2013): Chilean version of the INECO Frontal Screening (IFS-Ch): Psychometric properties and diagnostic accuracy. *Dementia & neuropsychologia*. 7:40-47.
65. Moreira HS, Lima CF, Vicente SG (2014): Examining Executive Dysfunction with the Institute of Cognitive Neurology (INECO) Frontal Screening (IFS): normative values from a healthy sample and clinical utility in Alzheimer's disease. *Journal of Alzheimer's Disease*. 42:261-273.
66. Fittipaldi S, Abrevaya S, de la Fuente A, Pascariello GO, Hesse E, Birba A, et al. (2020): A multidimensional and multi-feature framework for cardiac interoception. 212:116677.
67. Ekman P, Friesen WV (2003): *Unmasking the face: A guide to recognizing emotions from facial clues*. Ishk.
68. Rencher AC (2003): *Methods of multivariate analysis*. John Wiley & Sons.
69. Li Y, Nan B, Zhu J (2015): Multivariate sparse group lasso for the multivariate multiple linear regression with an arbitrary group structure. *Biometrics*. 71:354-363.
70. Dienes Z, Mclatchie NJPb, review (2018): Four reasons to prefer Bayesian analyses over significance testing. 25:207-218.
71. Kass RE, Raftery AEJJotasa (1995): Bayes factors. 90:773-795.
72. Lee MD, Wagenmakers E-J (2014): *Bayesian cognitive modeling: A practical course*. Cambridge university press.
73. Rouder JN, Morey RD, Speckman PL, Province JMJJomp (2012): Default Bayes factors for ANOVA designs. 56:356-374.
74. Rouder JN, Speckman PL, Sun D, Morey RD, Iverson GJPb, review (2009): Bayesian t tests for accepting and rejecting the null hypothesis. 16:225-237.
75. Morey RD, Rouder JN, Jamil T, Morey MRDJUhcr-pwpBBpi (2015): Package 'bayesfactor'.
76. Dobbin KK, Simon RMJBmg (2011): Optimally splitting cases for training and testing high dimensional classifiers. 4:1-8.
77. Lanka P, Rangaprakash D, Dretsch MN, Katz JS, Denney TS, Deshpande GJBi, et al. (2020): Supervised machine learning for diagnostic classification from large-scale neuroimaging datasets. 14:2378-2416.
78. Poldrack RA, Gorgolewski KJ, Varoquaux GJARoBDS (2019): Computational and informatic advances for reproducible data analysis in neuroimaging. 2:119-138.
79. Poldrack RA, Baker CI, Durnez J, Gorgolewski KJ, Matthews PM, Munafò MR, et al. (2017): Scanning the horizon: towards transparent and reproducible neuroimaging research. 18:115-126.
80. Chen T, Guestrin C (2016): Xgboost: A scalable tree boosting system. *Proceedings of the 22nd acm sigkdd international conference on knowledge discovery and data mining*, pp 785-794.
81. Zeng X, Luo GJHis, systems (2017): Progressive sampling-based Bayesian optimization for efficient and automatic machine learning model selection. 5:1-21.
82. Feurer M, Hutter F (2019): Hyperparameter optimization. *Automated machine learning*: Springer, Cham, pp 3-33.
83. Mason L, Baxter J, Bartlett P, Frean M (1999): Boosting algorithms as gradient descent in function space. *Proc NIPS*, pp 512-518.
84. Zheng H, Yuan J, Chen LJE (2017): Short-term load forecasting using EMD-LSTM neural networks with a Xgboost algorithm for feature importance evaluation. 10:1168.
85. Uddin S, Khan A, Hossain ME, Moni MAJBmi, making d (2019): Comparing different supervised machine learning algorithms for disease prediction. 19:1-16.
